# GPR65 Inactivation in Tumor Cells Drives Antigen-Independent CAR T-cell Resistance via Macrophage Remodeling

**DOI:** 10.1158/2159-8290.CD-24-0841

**Published:** 2025-02-25

**Authors:** Jayadev Mavuluri, Yogesh Dhungana, Lindsay L. Jones, Sheetal Bhatara, Hao Shi, Xu Yang, Song-Eun Lim, Noemi Reyes, Hongbo Chi, Jiyang Yu, Terrence L. Geiger

**Affiliations:** 1 Department of Pathology, St. Jude Children’s Research Hospital, Memphis, Tennessee.; 2 Department of Computational Biology, St. Jude Children’s Research Hospital, Memphis, Tennessee.; 3 Graduate School of Biomedical Sciences, St. Jude Children’s Research Hospital, Memphis, Tennessee.; 4 Department of Immunology, St. Jude Children’s Research Hospital, Memphis, Tennessee.; 5 College of Graduate Health Sciences, University of Tennessee Health Science Center, Memphis, Tennessee.

## Abstract

Chimeric antigen receptor (CAR) T-cell therapies, while promising for CD19^+^ hematologic malignancies, often face setbacks due to relapses. Our research identified G protein–coupled receptor 65 (GPR65) as a tumor-specific determinant affecting the efficacy of CAR T-cell therapy. In human patients and an immune-competent mouse model of B-cell acute lymphoblastic leukemia, low GPR65 expression correlates with resistance to CD19^+^ CAR T treatment. GPR65 knockout (GPR65 KO) tumors in mice similarly exhibit resistance. Through single-cell network analyses, we discovered that GPR65 deficiency reshapes tumor interactions with host macrophages by increasing tumor VEGFA levels, leading to macrophage expansion and preferential M2 polarization. Depleting host macrophages or deleting VEGFA in GPR65 KO tumors restores CAR T-cell therapy responsiveness. Moreover, combining anti-VEGFA therapy with CAR T-cell treatment significantly prolongs the survival of mice bearing GPR65 KO tumors. These findings emphasize the profound impact of tumor gene expression on the tumor microenvironment and subsequent CAR T-cell therapy outcomes.

**Significance:**

The study identifies GPR65 as an important determinant of B-cell acute lymphoblastic leukemia response to CAR T-cell therapy. Notably, GPR65 absence signals CAR T resistance. By emphasizing the therapeutic potential of targeting VEGFA or host macrophages, our study identifies routes to optimize CAR T-cell therapy outcomes in hematologic malignancies via tumor microenvironment manipulation.

## Teaser

Integrative and cross-species systems biology and *in vivo* modeling identifies tumor-intrinsic GPR65 as a regulator of macrophage-driven CAR T-cell resistance and a translational biomarker for CAR T-cell response in B-ALL.

## Introduction

Adoptively transferred T lymphocytes engineered to express chimeric antigen receptors (CAR) targeting CD19 are curative for some patients with relapsed or refractory B-cell acute lymphoblastic leukemia (B-ALL; refs. 1–4). Many patients, however, relapse or fail to respond after treatment. Identifying mechanisms underlying therapeutic resistance to CAR T-cell therapy is a necessary prelude to developing more effective approaches.

Preclinical and clinical studies seeking to augment CAR T-cell effectiveness have largely focused on CAR T-cell function. CAR T-cell exhaustion and the absence of long-term memory are associated with poor outcomes (5). Manipulations diminishing exhaustion and enhancing CAR T-cell memory can increase CAR T-cell effectiveness (6–8). The age and phenotype of patient-derived T cells used for CAR T-cell production and the quality and strength of signaling by CAR costimulatory domains influence these properties, and these insights have been incorporated into CAR designs and manufacturing paradigms (9–11).

Tumor features, including tumor heterogeneity, may also foster CAR T-cell resistance and poorer clinical outcomes (12). Most prominently, the outgrowth of CD19-negative or lineage-switched tumors is a common mechanism of CD19-specific CAR T resistance (13). However, tumors retaining the CAR ligand may also be resistant, and this is less studied. Tumor cells may alter CAR T-cell responses by promoting changes in the host immune system and tumor microenvironment (TME). Such TME changes are observed to mediate resistance in solid tumors (14) but are undefined and challenging to study in hematologic malignancies, such as B-ALL, in part because of the common use of immunodeficient mice or human–mouse graft systems that disrupt normal cellular interactions (15).

In this study, we use patient data and an immunocompetent mouse model of CD19-directed CAR T-cell therapy targeting heterogeneous B-ALL tumors to identify novel tumor-intrinsic factors affecting CAR T-cell therapeutic outcomes. We implicate tumor expression of the G protein–coupled receptor 65 (GPR65; TDAG8) in antigen-independent CAR T-cell resistance. Low GPR65 activity is associated with resistance to anti-CD19 CAR T-cell therapy in both patients and an immunologically intact mouse model independent of tumor CD19 expression. GPR65 is a proton-sensing cell surface receptor that stimulates cyclic AMP (cAMP) formation in response to acidic stimuli (16, 17). It has been previously identified as a tumor survival factor in some solid tumor cell lines although it can suppress tumor growth and survival in hematologic tumors (18, 19).

To dissect the role of GPR65 in CAR T resistance, we generated an immunocompetent GPR65 knockout (KO) and human CD19–expressing B-ALL mouse models. These are rendered wholly resistant to CAR T-cell therapy. Single-cell analyses reveal that this is mediated by alterations in the TME and associated with tumor-dependent host macrophage polarization from M1 to M2. GPR65-mediated suppression of tumor VEGFA production plays a prominent role. Depletion of host macrophages or VEGF blockade completely restores the sensitivity of GPR65 KO tumor–bearing mice to anti-CD19 CAR T-cell therapy. Our findings demonstrate the exquisite sensitivity of adoptive immunotherapy to a single tumor–expressed gene through tumor–TME interactions and TME reprogramming. GPR65 expression may function as a predictive biomarker for response of patients with B-ALL to CD19 CAR T-cell therapy. Our work further highlights potential approaches to overcome B-ALL resistance to CAR T-cell therapy through TME manipulation.

## Results

### B-ALL GPCR Signaling Predicts Preclinical Responses to CAR T-cell Therapy

We sought novel tumor-intrinsic determinants of tumor resistance to CAR T-cell therapy using an immunocompetent model of B-ALL in which human CD19 (hCD19)–transduced, mouse BCR1-ABL^+^ pre–B-ALL tumor clones were transferred into unconditioned C56BL/6 mice (7, 20). Following tumor engraftment, the mice were treated with activated CAR T cells isolated from CD19-CAR transgenic mice (7).

**Figure 1.**
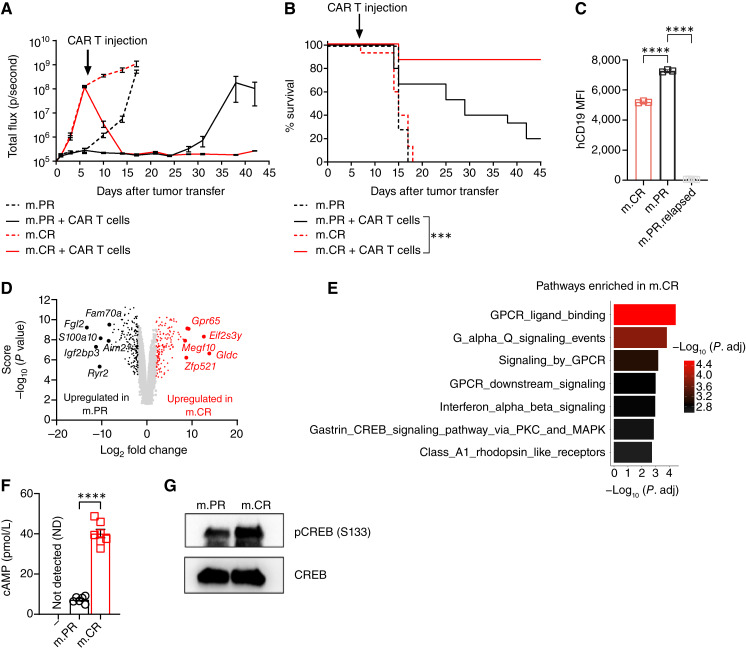
B-ALL GPCR signaling predicts preclinical responses to CAR T-cell therapy. **A** and **B,** Mice received 1 × 10^6^ m.PR or m.CR tumor cells (day 0) and 10 × 10^6^ CAR T cells or PBS 7 days later. **A,***In vivo* tumor bioluminescence imaging. Representative of three experiments, with *n* = 5 mice per group. **B,** Kaplan–Meier survival. Statistical significance was calculated using the log-rank (Mantel–Cox) test. Pooled from three experiments, *n* = 7 (m.PR); *n* = 15 (m.PR + CAR-T, m.CR, m.CR + CAR-T) mice per group. **C,** Surface expression of human CD19 on m.PR, m.CR, and m.PR relapsed tumor cells from *in vitro* culture or *in vivo* after relapse (day 24–35). Significance determined by one-way ANOVA with the Tukey *post hoc* test for multiple comparisons. MFI, mean fluorescence intensity. **D** and **E,** RNA-seq of *in vitro* cultured m.PR and m.CR tumor clones. *n* = 3 per group. **D,** Volcano plot highlighting top differentially expressed genes comparing m.CR and m.PR tumor clones. **E,** Functional enrichment analysis of upregulated differentially expressed genes in m.CR vs. m.PR tumors using REACTOME pathway gene sets (MsigDB v7.4). Pathways were filtered at Benjamini–Hochberg-corrected *P* value < 0.01. **F,** cAMP concentration in m.PR and m.CR tumor cells or media control, with *n* = 6 per group. Statistical significance was calculated using an unpaired *t* test. **G,** Immunoblots of pCREB and total CREB protein levels in m.PR and m.CR tumor cells. Representative of two experiments. All error bars represent mean ± SEM. ***, *P* < 0.001; ****, *P* < 0.0001.

We observed that two separately derived BCR1-ABL^+^ hCD19^+^ B-ALL clones (“Methods”) demonstrated distinct responses to CAR T-cell treatment: partial response (m.PR) and complete response (m.CR). m.CR tumors grew aggressively but responded completely to CAR T-cell treatment, with little relapse; however, m.PR tumors exhibited slow growth and initial response to CAR T-cell treatment but relapsed and succumbed to the tumor (Fig. 1A and B). Although both tumor types initially expressed high levels of hCD19, the relapsed m.PR tumors lost hCD19 expression (Fig. 1C).

Comparative analysis of the m.PR and m.CR tumor cells at baseline by RNA sequencing (RNA-seq) identified the G protein–coupled receptor (GPCR) *Gpr65* (*Tdag8*) among the top upregulated genes in CR tumors (Fig. 1D; Supplementary Table S1). Indeed, GPCR signaling and the pathways associated with it (GPCR ligand binding, G protein–coupled receptor signaling, GPCR downstream signaling, G-alpha signaling events, and cAMP-responsive element binding protein (CREB) signaling via PKC and MAPK) were among the pathways identified as enriched in m.CR compared with m.PR tumor cells by functional enrichment analysis (Fig. 1E; Supplementary Table S2). GPR65 promotes cAMP formation, which mediates activation of the transcription factor (TF) CREB (16, 21, 22). Consistently, m.CR tumors had increased cytosolic cAMP and CREB S133 phosphorylation relative to m.PR cells (Fig. 1F and G).

### Cross-species Integrative Analysis Ranks GPR65 as the Top CAR T Response Driver in B-ALL

To determine the relevance of GPR65 in human tumor responses, we analyzed data from a clinical trial of CD19 CAR T treatment of patients with B-ALL (23) and patients treated with blinatumomab (24). These analyses profiled patient tumor samples and had a sufficient sample size in responder and nonresponder groups to identify tumor-response correlates. In the CD19 CAR T treatment cohort, 19 responders had durable (>1 year) complete responses (h.CR), and eight nonresponders (h.NR) failed to respond and had no evidence of CD19 antigen loss. In the blinatumomab cohort, 19 patients responded, and 20 patients did not respond to blinatumomab engager therapy.

**Figure 2.**
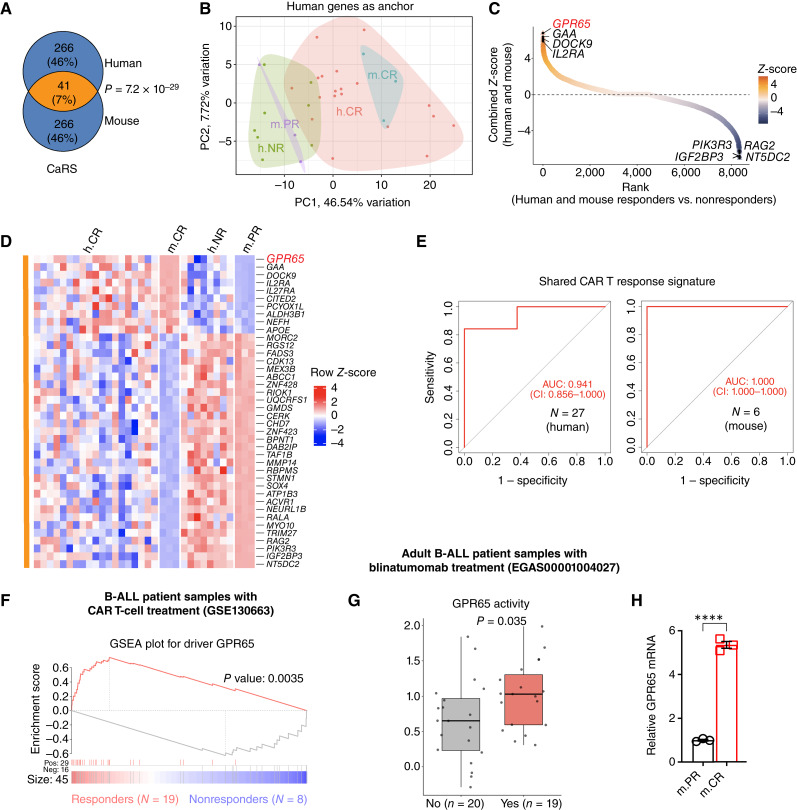
Cross-species integrative analysis ranks GPR65 as the top CAR T response driver in B-ALL. **A,** Venn diagram of the CaRS derived independently by comparing h.CR (responders, *n* = 19) and h.NR (nonresponders, *n* = 8) from bone marrow RNA-seq profiles of patients and RNA-seq of *in vitro* cultured mouse m.PR and m.CR tumor cells (*n* = 3 per group). Statistical significance of overlap computed using the two-tailed Fisher exact test. **B,** Principal component analysis plot integrating human and mouse RNA-seq profiles performed using the expression of 307 human CaRS genes. PC, principal component. **C,** S-curve of combined gene ranking using a total of 8,116 genes for human (h.CR vs. h.NR) and mouse (m.CR vs. m.PR; responders vs. nonresponders). The top four upregulated and downregulated genes are highlighted, and the top hit GPR65 is highlighted in red. **D,** Gene expression heatmap of 41 genes shared between human and mouse CaRS in both patient and mouse responders (h.CR and m.CR) and nonresponders (h.NR and m.PR) ranked by combined *Z*-score from **C**. GPR65 is highlighted in red. **E,** ROC curve plot for evaluation of the shared CaRS (41 genes) score to predict the response of patients with B-ALL and B-ALL mouse to CD19 CAR T-cell therapy. CI, confidence interval. **F,** GSEA plot of GPR65 targets from bone marrow RNA-seq profiles of B-ALL patient responders vs. nonresponders treated with CD19 CAR T cells. Responders (*N* = 19) are defined as patients who had durable (>1 year) complete remissions, and nonresponders (*N* = 8) are defined as patients who did not respond to treatment and had no evidence of CD19 antigen loss. *P* value was estimated using the Wilcoxon rank-sum test. **G,** Boxplot of GPR65 activity calculated using the NetBID2 algorithm using RNA-seq profiles of adult patients with B-ALL treated with blinatumomab. Responders (*N* = 19) are patients who achieved complete remission by morphology using standard International Working Group criteria, and nonresponders (*N* = 20) are patients who did not respond to blinatumomab therapy and had no evidence of CD19 antigen loss. *P* value was estimated using the Wilcoxon rank-sum test. Boxplot shows a summary of the data interims of the minimum, maximum, sample median, and the first and third quartiles. **H,** qRT-PCR analysis showing relative *Gpr65* levels in m.PR and m.CR tumor cells. Representative of three experiments. Statistical significance was calculated using an unpaired *t* test. All error bars represent mean ± SEM. ****, *P* < 0.0001.

We assessed whether our m.PR and m.CR B-ALL clones mirrored the human patient tumors by selecting CAR T response signature (CaRS) genes from both species and comparing responder and non-responder groups using a three-step strategy (“Methods”). Human and mouse CaRS significantly overlapped (Fig. 2A), with principal component analysis distinguishing CAR T therapy responders from nonresponders in both species (Fig. 2B; Supplementary Fig. S1A). Subsequent integrative analysis of differentially expressed genes in humans and mice ranked *GPR65* as the top candidate gene linked with responders in both species (Fig. 2C). A heatmap showcasing shared genes within the CaRS confirmed that m.PR and m.CR B-ALL tumor cells parallel human patient tumor samples (Fig. 2D). ROC analysis using the shared genes within the CaRS indicated that the gene signatures derived from m.PR and m.CR B-ALL, together with h.CR and h.NR, stratified CD19 CAR T therapy patients with 94% accuracy (Fig. 2E). Furthermore, consistent with the findings from Singh and colleagues (23), downregulation of death receptor genes was observed in m.PR tumors compared with that in m.CR tumors (Supplementary Fig. S1B), indicating that the impaired death receptor phenotype identified in human B-ALL tumors resistant to CAR T-cell therapy is conserved in our murine model.

To overcome the potential issues arising from small sample size and interpatient heterogeneity, we applied a data-driven, network-based hidden driver inference algorithm (NetBID2; refs. 25, 26) to analyze the pretreatment tumor RNA-seq dataset (Supplementary Fig. S1C). First, we used the SJARACNe algorithm (27) to reverse-engineer a gene–gene interactome specific to B-ALL from RNA-seq profiles of 1,988 patients with B-ALL. Next, we superimposed the B-ALL–specific interactome on the bulk RNA-seq profiles of pretreated B-ALL tumors in the CAR T clinical study and transformed the transcriptomic profiles into activity profiles of 1,024 TFs and 6,349 signaling factors. As part of the B-ALL interactome, the GPR65 subnetwork (Supplementary Fig. S1D) consisted of 50 regulon genes, including 30 positive and 20 negative targets. Functional enrichment highlighted the role of the GPR65 regulon in innate and adaptive immune responses (Supplementary Fig. S1E). Gene set enrichment analysis (GSEA) revealed that positive targets of the GPR65 regulon were significantly enriched in responders and negative targets in nonresponders (Fig. 2F). GPR65 activity and expression were also significantly higher in tumors isolated from responders (*n* = 19) than in nonresponders (*n* = 8; Supplementary Fig. S1F). Moreover, ROC analysis of GPR65 activity and expression demonstrates its predictive power for CAR T therapy response in patients (Supplementary Fig. S1G). In addition, we observed similar upregulation of GPR65 expression and activity in adult patients with B-ALL responding to blinatumomab engager without the loss of CD19 expression (Fig. 2G; Supplementary Fig. S1H and S1I). These findings provide evidence that GPR65-dependent immunotherapy resistance extends to adult B-ALL and alternative immunotherapies. We validated *GPR65* expression in our m.PR and m.CR B-ALL clones, with m.CR clones expressing significantly higher *Gpr65* compared with m.PR clones (Fig. 2H).

### GPR65 KO Tumors Drive CAR T Resistance *In Vivo* without Reducing Cognate Antigen Expression or CAR T-cell Expansion

To further investigate the role of GPR65 in tumor responses to CAR T-cell therapy, we knocked out *Gpr65* in the m.CR hCD19^+^ B-ALL cell line. Four single-guide RNAs were used to delete *Gpr65* exon 2 (GPR65 KO1). Alternatively, a single single-guide RNA was used to generate three clones (GPR65 KO2, GPR65 KO3, and GPR65 KO4; GPR65 KO pool) with insertions or deletions disrupting translation (Supplementary Fig. S2A and S2B). GPR65 KO1 was also transduced with GPR65-IRES-MSCV retrovirus to generate a clone with GPR65 overexpression. Effective knockout and overexpression of GPR65 were confirmed through analysis of GPR65 mRNA expression, CREB phosphorylation, and cAMP production (Supplementary Fig. S2C–S2E).

**Figure 3.**
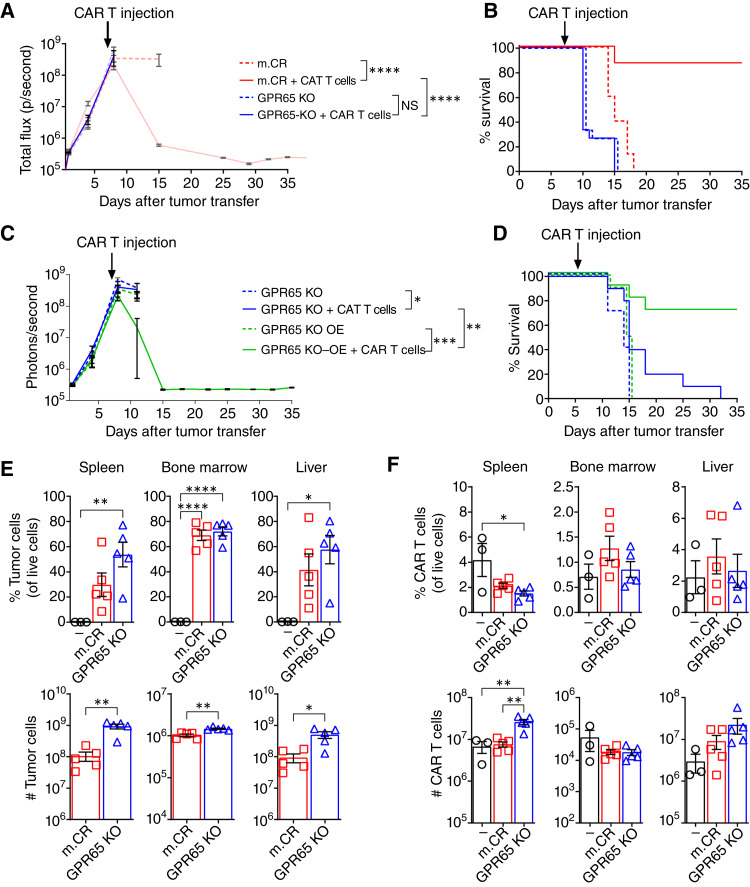
GPR65 KO tumors drive CAR T resistance *in vivo* without reducing cognate antigen expression or CAR T-cell expansion. **A** and **B,** Mice received 1 × 10^6^ m.CR or GPR65 KO tumor cells on day 0, followed by treatment with 10 × 10^6^ CAR T cells or PBS 7 days later. **A,** Bioluminescence imaging showing tumor growth. Representative of three experiments, with *n* = 5 mice per group. **B,** Kaplan–Meier survival curves. Statistical significance was calculated using the log-rank (Mantel–Cox) test. Pooled from three experiments, *n* = 15 mice per group. **C** and **D,** Mice received 1 × 10^6^ GPR65 KO1 or GPR65 KO1 OE tumor cells, followed by treatment with 10 × 10^6^ CAR T cells or PBS 7 days later. **C,** Bioluminescence imaging showing tumor growth. Representative of two experiments, with *n* = 5 mice per group. OE, overexpression. **D,** Kaplan–Meier survival curves. Significance was determined using the log-rank (Mantel–Cox) test. Pooled from two experiments, with *n* = 10 mice per group. **E** and **F,** Spleen, bone marrow, and liver were harvested 4 days after CAR T treatment, and tumor and CAR T cells were analyzed by flow cytometry. **E,** Tumor cell percentage and number. **F,** CAR T-cell percentage and number. Representative of three experiments, with *n* = 3–5 mice per group. Significance was determined by one-way ANOVA with the Tukey *post hoc* test for multiple comparisons. Error bars represent mean ± SEM. *, *P* < 0.05; **, *P* < 0.01; ***, *P* < 0.001; ****, *P* < 0.0001.

We next assessed GPR65 KO tumor response to anti-CD19 CAR T-cell therapy. No difference in *in vitro* expansion was observed between m.CR and GPR65 KO cells (Supplementary Fig. S3A). Further, hCD19 antigen expression on m.CR and GPR65 KO cells displayed no difference at both basal level and 7 days after tumor injection (Supplementary Fig. S3B). In our *in vivo* model, m.CR and GPR65 KO tumors showed similar growth rates, with similar tumor burden at the time of CAR T-cell treatment (Fig. 3A). However, unlike the complete regression seen with m.CR tumors, GPR65 KO tumors showed no response to CAR T-cell treatment or survival advantage relative to untreated mice (Fig. 3A and B). Similar results were observed after transferring the GPR65 KO pool and CAR T-cell treatment (Supplementary Fig. S3C and S3D). In contrast, overexpression of GPR65 in GPR65 KO tumors rescued responsiveness to CAR T-cell treatment *in vivo* (Fig. 3C and D). These data indicated that GPR65 deficiency drives antigen-independent B-ALL tumor refractoriness to CAR T-cell therapy *in vivo*.

One established cause of CAR T-cell therapy failure is the inability of CAR T cells to expand and persist *in vivo* (28). We therefore quantified tumor and CAR T-cell numbers in mice with no tumor or m.CR or GPR65 KO tumors 4 days after CAR T treatment. Mice with GPR65 KO tumors had a higher tumor burden in all organs examined than mice with m.CR tumors (Fig. 3E; Supplementary Fig. S3E), and this was associated with increased tumor-associated morbidities and diminished survival. Consistent with Supplementary Fig. S1B, we observed a similar downregulation of nine of 15 detected death receptor genes in GPR65 KO tumors compared with m.CR tumors (Supplementary Fig. S3F). CAR T cells in mice bearing GPR65 KO tumors were also present in greater or equal numbers compared with those in mice with m.CR tumors (Fig. 3F). These results indicated that the failure of GPR65 KO tumors to respond to treatment was not due to diminished CAR T-cell survival or expansion in tumor-bearing organs.

### Preserved Effector Function in CAR T Cells from Mice with GPR65 KO Tumors

**Figure 4.**
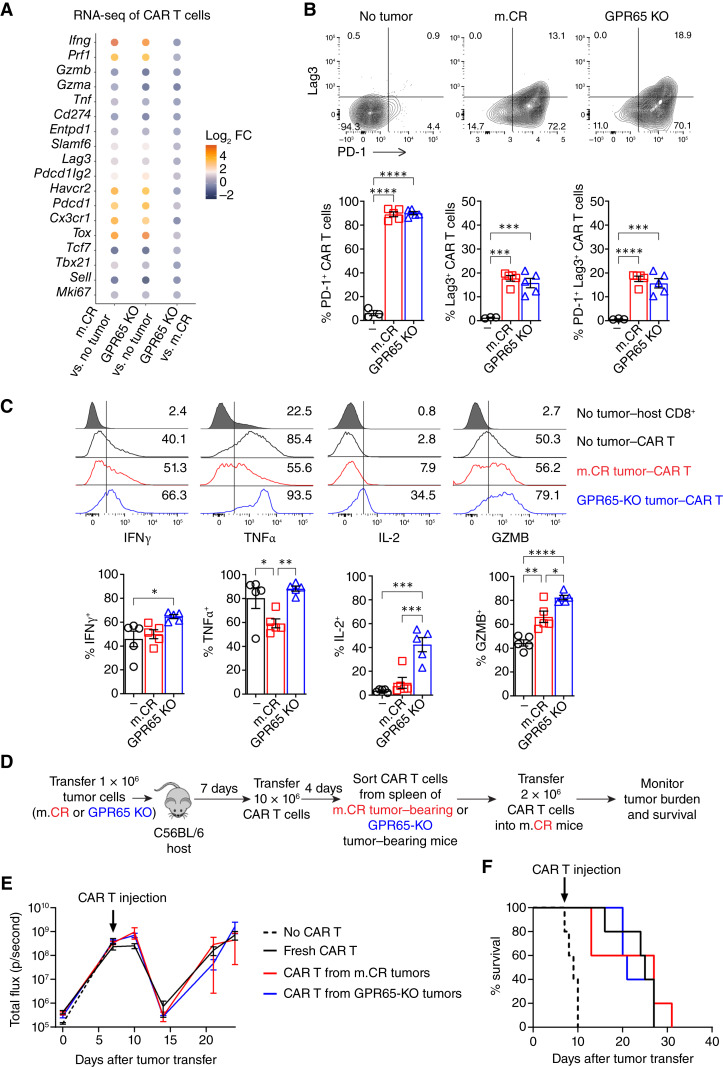
CAR T cells from mice with GPR65 KO tumors retain effector functions. **A,** Bubble plot with color showing fold change in expression of T-cell exhaustion and effector genes from CAR T cells isolated from mice with no tumor or m.CR or GPR65 KO tumors. *n* = 2–3 replicates per group. Log_2_FC, log_2_ fold change. **B,** Representative flow cytometry plots and summary graphs of PD-1 and Lag3 expression on CAR T cells from mice with no tumor or with m.CR or GPR65 KO tumors; *n* = 3–5 mice per group. Significance was determined by one-way ANOVA with the Tukey *post hoc* test for multiple comparisons. **C,** Percentage of CAR T cells expressing IFNγ, TNFα, IL-2, and GZMB in the spleen of CAR T–treated mice. Representative of three experiments, with *n* = 3–5 mice per group. Significance was determined by one-way ANOVA with the Tukey *post hoc* test for multiple comparisons. CAR T cells were activated for 7 days before transfer into tumor-bearing mice. The cells were isolated 4 days after transfer, restimulated with phorbol 12-myristate 13-acetate and ionomycin *ex vivo*, and stained intracellularly for cytokines. **D,** Schematic of the CAR T cell retransfer experiment. **E** and **F,** Mice received 1 × 10^6^ m.CR tumor cells on day 0, followed by treatment with 2 × 10^6^ fresh CAR T cells or CAR T cells isolated from m.CR or GPR65 KO tumor–engrafted mice 7 days later. **E,** Bioluminescence imaging showing tumor growth. Representative of two experiments, with *n* = 5 mice per group. **F,** Kaplan–Meier survival curves. Statistical significance was calculated using the log-rank (Mantel–Cox) test. Representative of two experiments, with *n* = 5 mice per group. All error bars represent mean ± SEM. *, *P* < 0.05; **, *P* < 0.01; ***, *P* < 0.001; ****, *P* < 0.0001.

To evaluate exhaustion and effector functions (29) in the tumor-infiltrating CAR T cells *ex vivo* (after restimulation with phorbol 12-myristate 13-acetate (PMA) and ionomycin), we first performed RNA-seq of isolated CAR T cells from mice without tumors or mice bearing m.CR or GPR65 KO tumors, followed by differential expression (DE) and pathway analysis. Relative to tumor-free mice, CAR T cells from mice with either m.CR or GPR65 KO tumors upregulated genes associated with exhaustion. However, no significant difference in the expression of these genes was seen in CAR T cells from mice with the two tumor types (Fig. 4A; Supplementary Fig. S4A; Supplementary Table S3). GSEA revealed enrichment of effector and CXCR5^+^ signatures in CAR T cells from GPR65 KO tumors (Supplementary Fig. S4B) previously described to be generated because of antigen persistence (30). Consistently, surface expression of the inhibitory receptors PD-1 and LAG3 was elevated although it did not differ in CAR T cells from the m.CR and GPR65 KO groups (Fig. 4B). These cells likewise showed similar effector and memory cell proportions based on CD44 and CD62L staining (Supplementary Fig. S4C).

Relative to CAR T cells from mice with m.CR tumors, CAR T cells from GPR65 KO tumors showed equal or increased expression of IFNγ, TNFα, IL-2, and granzyme B (GZMB), indicating preservation of functional potential despite their lack of therapeutic efficacy (Fig. 4C). Similarly, CAR T cells from GPR65 KO tumors showed elevated expression of activation markers CD25 and CD69, consistent with the increased tumor burden and antigen exposure (Supplementary Fig. S4D and S4E).

To determine whether the CAR T retained antitumor activity against m.PR, m.CR, or GPR65 KO tumors *in vitro*, co-culture killing and repetitive stimulation assays were performed, followed by measurement of surface expression of activation markers (CD25 and CD69), terminal differentiation marker (KLRG1), and inhibitory markers (PD-1, TIM3, and LAG3) by flow cytometry and secreted cytokines (IFNγ, TNFα, and GZMB) by ELISA (Supplementary Fig. S4F–S4I). We did not observe differences in *in vitro* antitumor activity of CAR T cells against these different tumors. Next, we isolated transferred CAR T cells 4 days after treatment of m.CR or GPR65 KO tumor–bearing mice and then retransferred these T cells into new mice with established m.CR tumors (Fig. 4D). Due to the limited numbers of isolated CAR T cells, a reduced number (one fifth) of therapeutic cells was administered in these secondary transfers. An additional control group received the same number of fresh, *in vitro*–activated CAR T cells. All groups of CAR T cells equivalently reduced tumor burden and increased survival of the m.CR tumor-bearing recipients (Fig. 4E and F). Although all mice experienced relapse, likely because of decreased transferred CAR T-cell numbers, there was no significant difference in tumor burden or survival. To confirm that the CAR T cells from GPR65 KO–grafted mice retain functionality after treatment, a rechallenge experiment was performed in mice receiving m.CR tumors. This demonstrated preserved function of CAR T cells after tumor rechallenge (Supplementary Fig. S4J).

The above results showed that CAR T cells from GPR65 KO and m.CR tumors retained effector functionality despite their inability to clear the GPR65 KO tumors and indicated that GPR65 KO tumor cells did not confer CAR T resistance through downregulation of CD19 expression or impairment of CAR T-cell expansion and function. Further, all tumors (m.PR, m.CR, and GPR65 KO) were equally susceptible to CAR T killing *in vitro*. This suggested that alterations in the TME may have mediated CAR T resistance to GPR65 KO tumors and led to the hypothesis that tumor cells rewired tumor–TME interactions, leading to the local ineffectiveness of CAR T cells.

### scRNA-seq Characterization of m.CR and GPR65 KO Tumors and TME

**Figure 5.**
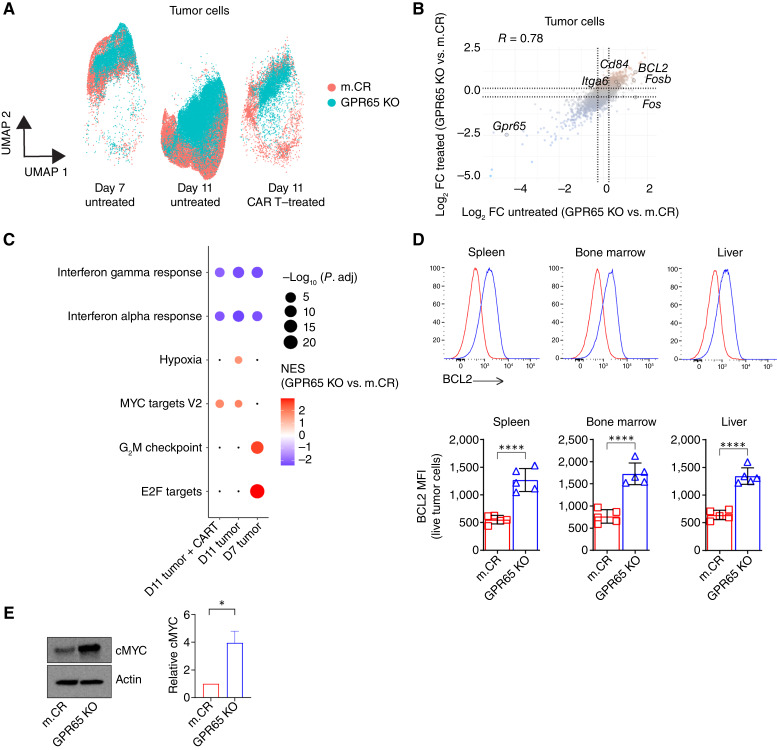
scRNA-seq characterization of mouse CR and GPR65 KO tumors. **A,** Uniform Manifold Approximation and Projection (UMAP) plot showing m.CR and GPR65 KO tumor populations from mice prior to CAR T-cell treatment (days 7 and 11) and after CAR T-cell therapy (day 11). **B,** Scatter plot highlighting key differentially expressed genes in CAR T cell–treated and untreated GPR65 KO vs. m.CR tumors. The *x*-axis represents log_2_ fold difference (log_2_FC) in gene expression comparing untreated GPR65 KO vs. m.CR tumors. The *y*-axis represents the log_2_ fold difference in gene expression comparing CAR T cell–treated GPR65 KO vs. m.CR tumors. The dotted line represents a log_2_ fold change cutoff at 0.25. Representative of scRNA-seq performed using five mice in each group. **C,** Bubble plots showing enrichment of statistically significant Hallmark pathways from MsigDB (v7.5.1) for day 7 (D7) untreated, day 11 untreated, and day 11 (D11) CAR T–cell treated GPR65 KO vs. m.CR tumors. Red represents normalized enrichment score (NES) in GPR65 KO tumors, and blue represents NES in m.CR tumors. The size of the bubble represents −log_10_ Benjamini–Hochberg-corrected *P* value, and black dots represent pathways that did not pass the statistical significance cutoff of *P* < 0.05. Representative of scRNA-seq performed using five mice in each group. **D,** Representative histograms and summary graphs showing expression of BCL2 in m.CR (red) or GPR65 KO (blue) tumors isolated from mice 4 days after CAR T-cell treatment. Representative of three experiments, with *n* = 5 mice per group. Significance was determined using an unpaired *t* test. MFI, mean fluorescence intensity. **E,** Immunoblots showing cMYC and actin protein levels in m.CR or GPR65 KO tumor cell lysates from culture (left) and their quantification (right). Representative of two experiments. All error bars represent mean ± SEM. *, *P* < 0.05; ***, *P* < 0.0001.

We next employed single-cell RNA-seq (scRNA-seq) to resolve potential tumor and TME mechanisms of CAR T resistance. GPR65 KO or m.CR tumors [red fluorescent protein–positive (RFP^+^)] and host immune cells (CD45.2^+^ Thy1.1^−^ RFP^−^) were isolated from the spleen of tumor-bearing mice prior to CAR T-cell treatment (day 7) and 4 days after treatment (day 11) and analyzed (Supplementary Fig. S5A). We performed InferCNV (31–34) analysis to annotate tumor cells (Supplementary Fig. S5B) and separated tumor cells from the rest of the TME. Clustering of tumor cells revealed that GPR65 KO and m.CR tumors were distinct with further divergence after CAR T-cell treatment (Fig. 5A; Supplementary Fig. S5C). DE analysis of the tumors demonstrated several differences. GPR65 KO tumors expressed increased TFs of the AP-1 family (*Fos* and *Fosb*) and the antiapoptotic marker *Bcl2*, known to promote tumor cell survival (35, 36). Cell surface genes *Itga6* and *Cd84*, described as conferring adhesion-mediated drug resistance and an immunosuppressive microenvironment, were also upregulated by GPR65 KO tumors (Fig. 5B; Supplementary Fig. S5D; Supplementary Table S4; refs. 37, 38). GSEA revealed that GPR65 KO tumors were enriched in gene signatures associated with G_2_M checkpoints, E2F targets, and MYC targets (Fig. 5C; Supplementary Fig. S5E), which are also associated with proliferation and survival (39). In addition, GPR65 KO tumors exhibited downregulation of genes associated with interferon and inflammatory responses, and this has been linked to immune evasion in solid malignancies (Fig. 5C; Supplementary Fig. S5F; ref. 40).

Consistent with scRNA-seq analysis, BCL2 protein was increased in GPR65 KO tumors from CAR T cell–treated mice by flow cytometry (Fig. 5D), and MYC was elevated at the protein level but not at mRNA (Fig. 5E; Supplementary Fig. S5D). Despite these differences, GPR65 KO tumor cells remained equally susceptible to CAR T cell–mediated lysis *in vitro* as m.CR tumors, even at low effector–to–target ratios, indicating that although scRNA-seq demonstrated nonidentity with m.CR tumors, GPR65 KO tumors are not intrinsically resistant to CAR T cell–mediated lysis.

### Macrophage Expansion and M1 to M2 Switch Are Seen in Humans and Mice and Confer CAR T Resistance

**Figure 6.**
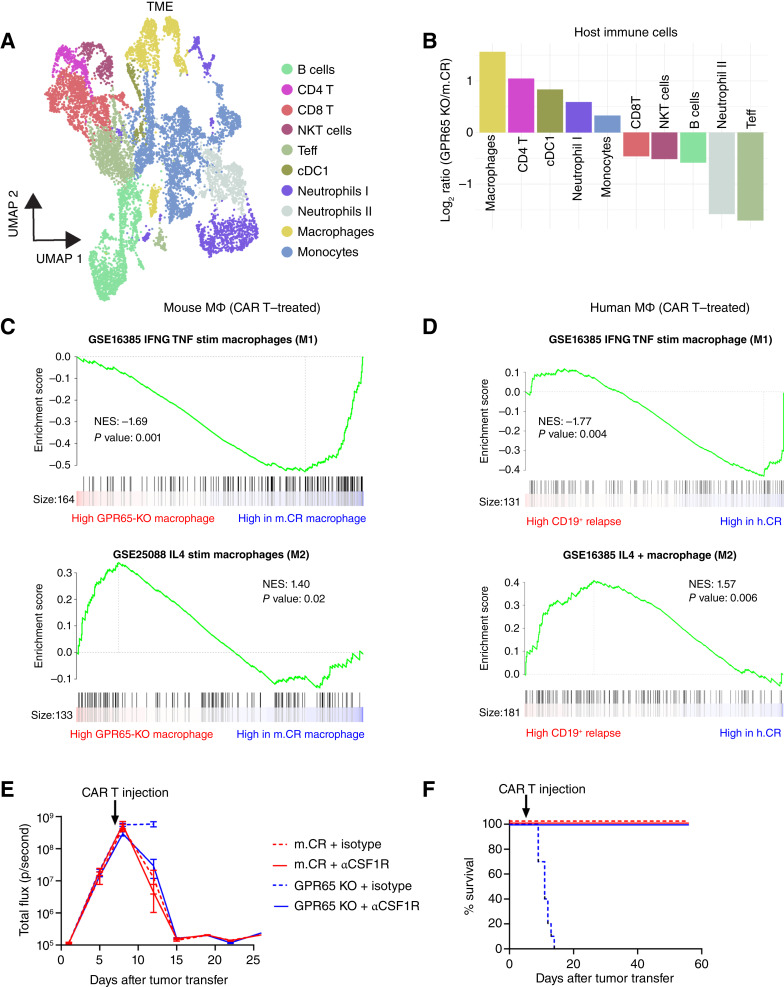
GPR65 KO tumors reprogram macrophages with expansion and an M1 to M2 switch that confers CAR T resistance. **A,** scRNA-seq Uniform Manifold Approximation and Projection (UMAP) plot showing host immune cell populations from the TME of CAR T–treated mice bearing m.CR and GPR65 KO tumors. Representative of scRNA-seq of tumor and TME performed at a tumor/TME ratio of 1:1 (CAR T cell–treated GPR65 KO or m.CR tumor–bearing mice). **B,** Waterfall plot of the log_2_ ratio of indicated GPR65 KO vs. m.CR host TME populations. **C,** GSEA plot of IFNγ- and TNF-stimulated macrophage signatures (M1; top) and GSEA plot of IL4-stimulated macrophage signatures (M2; bottom) comparing macrophages in GPR65 KO tumor–engrafted and m.CR tumor–engrafted mice after CAR T treatment. *P* value is calculated using the Kolmogorov–Smirnov test. Representative of scRNA-seq of tumor, F4/80^+^ enriched macrophages, and nonmacrophage TME cells at a ratio of 1:3:2. **D,** GSEA plot of IFNγ- and TNF-stimulated macrophage signatures (M1; top) and of IL4-stimulated macrophage signatures (M2; bottom) comparing CD68^+^ macrophages from CD19^+^ relapse and h.CR patients’ bone marrow TME after CAR T treatment. *P* value is calculated using the Kolmogorov–Smirnov test. NES, normalized enrichment score. **E,** Bioluminescence imaging showing tumor growth in CAR T–treated mice depleted of macrophages (αCSF1R) or treated with isotype control. Representative of two experiments, with *n* = 5 mice per group. **F,** Kaplan–Meier survival curves pooled from two experiments, with *n* = 10 mice per group. Statistical significance was calculated using the log-rank (Mantel–Cox) test. Teff, T effector; cDC, Classical dendritic cells. All error bars represent mean ± SEM.

To explore TME alterations, we examined the scRNA-seq data for the composition of host immune cell populations (Fig. 6A; Supplementary Fig. S6A and S6B). Differences in macrophage populations were particularly pronounced, with a 2.96-fold increase in CAR T–treated mice bearing GPR65 KO compared with m.CR tumors (Fig. 6B). This increase was confirmed by quantitative flow cytometry (Supplementary Fig. S6C). Macrophages can potentially generate an immunosuppressive TME, and this was further explored (41).

To validate our findings, we enriched F4/80^+^ macrophages before and after CAR T treatment followed by scRNA-seq (Supplementary Fig. S5A). We observed an adaptive response with expansion of the macrophage population induced by CAR T treatment in both m.CR and GPR65 KO tumor–engrafted mice. The GPR65 KO group experienced a significantly higher macrophage burden (Supplementary Fig. S6D). scRNA-seq revealed a similar observation and further highlighted the presence of heterogeneity in macrophages from the TME of these tumors (Supplementary Fig. S6E). In addition, macrophages from m.CR-engrafted mice expressed inflammatory cytokines such as *Tnf* and *Il1b*, which were retained after CAR T treatment, whereas macrophages from GPR65 KO–engrafted mice expressed *Chil3*, a known marker of anti-inflammatory macrophages (Supplementary Fig. S6F). To unbiasedly explore the macrophage state, we performed GSEA of macrophages using macrophage gene signatures stimulated by *Tnf*, *Ifnγ* (M1), or *Il4* (M2). Macrophages from m.CR–engrafted tumors displayed significant enrichment of an M1-like phenotype, whereas macrophages from GPR65 KO–engrafted tumors were enriched for an M2-like phenotype (Fig. 6C). To correlate our findings in patients undergoing CD19 CAR T-cell therapy, we used scRNA-seq profiles of unsorted bone marrow from three h.CR and two CD19^+^ relapse patients (43). We subset human bone marrow macrophages using *CD14* and *CD68* and performed GSEA. Consistently, macrophages from h.CR patients displayed an M1-like phenotype, whereas macrophages from CD19^+^ relapse patients’ bone marrow were enriched for an M2-like phenotype (Fig. 6D). Tumor-associated macrophages are known to confer an M2 polarization state, resulting in an immunosuppressive microenvironment (44–47). Our results suggest that the expanded, M2-polarized macrophages may be a common mechanism of primary resistance to CAR T-cell therapy in both mouse and human tumors.

To determine the influence of the expanded and polarized macrophages present in GPR65 KO mice *in vivo*, we depleted macrophages with the anti-CSF1R (CD115) antibody prior to CAR T therapy (Supplementary Fig. S7A). Macrophage, but not tumor, depletion was confirmed in the spleen and bone marrow prior to CAR T treatment (Supplementary Fig. S7B and S7C). As expected, mice bearing GPR65 KO tumors that received the isotype control antibody did not respond to CAR T-cell therapy, and similarly treated m.CR mice attained complete remission. However, macrophage-depleted mice with either m.CR or GPR65 KO tumors responded equivalently and fully to CAR T-cell therapy; complete remission with no tumor relapse was seen even after the depletion regimen was ended (Fig. 6E and F). Therefore, the expanded, M2-polarized macrophage pool in the GPR65 KO TME was necessary for CAR T-cell therapy resistance; eliminating macrophages restored CAR T-cell tumoricidal activity.

### GPR65 KO–Derived VEGFA Fosters CAR T-cell Therapy Resistance

**Figure 7.**
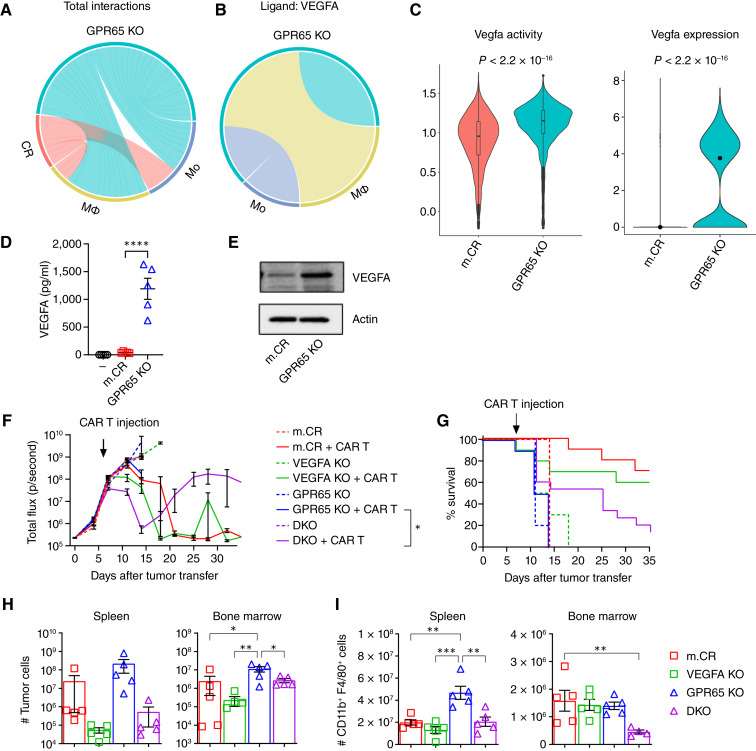
GPR65 KO–derived VEGFA fosters CAR T-cell therapy resistance. **A,** Chord diagram of cell–cell communication analysis performed using scRNA-seq data to quantify total interactions among m.CR tumors or GPR65 KO tumors, macrophages, and monocytes mediated by expressed ligands. Representative of scRNA-seq of tumor and TME performed at a tumor/TME ratio of 1:1 (CAR T cell–treated GPR65 KO tumor–bearing or m.CR tumor–bearing mice). **B,** Chord diagram showing GPR65 KO tumor–derived VEGFA ligand communication with host macrophages and monocytes. **C,** scRNA-seq analysis of VEGFA activity (left) and expression (right) in m.CR or GPR65 KO tumor cells isolated from mice 4 days after CAR T-cell treatment. Statistical significance was calculated using a two-tailed *t* test. **D,** Serum VEGFA concentrations in mice bearing no tumor, m.CR tumor, or GPR65 KO tumor 4 days after CAR T-cell treatment. Representative of two independent experiments. Significance was determined using one-way ANOVA with the Tukey *post hoc* test for multiple comparisons. **E,** Immunoblots showing VEGFA and actin protein levels in m.CR- or GPR65 KO–cultured tumor cell lysates. Representative of two experiments. **D** and **F–I,** Mice received 1 × 10^6^ of the indicated tumor cells, followed by treatment with 10 × 10^6^ CAR T cells 7 days later. **F,** Bioluminescence imaging showing tumor growth. Representative of two experiments, with *n* = 5 mice per group. **G,** Kaplan–Meier survival curves. Statistical significance was calculated using the log-rank (Mantel–Cox) test. Pooled from two experiments; *n* = 10 mice per group. **H,** Tumor and (**I**) macrophage cell numbers in the spleen and bone marrow of mice bearing the indicated tumor, 4 days after CAR T-cell treatment. Representative of two experiments, with *n* = 3–5 mice per group. Significance was determined using one-way ANOVA with the Tukey *post hoc* test for multiple comparisons. All error bars represent mean ± SEM. *, *P* < 0.05; **, *P* < 0.01; ***, *P* < 0.001; ****, *P* < 0.0001.

To probe for the mechanism by which GPR65 KO tumors modify the host macrophage pool, we performed cell–cell communication analysis using the LIANA ensemble ligand–receptor database (48) and CellChat (49). scRNA-seq data from CAR T cell–treated splenocytes were interrogated for ligand–receptor pairs in tumors, macrophages, and monocytes. GPR65 KO tumors displayed increased communications with host macrophages and monocytes compared with m.CR tumors (Fig. 7A). Although multiple ligand–receptor channels were identified linking GPR65 KO tumors but absent in m.CR tumors, a particular gain was observed for *Vegfa* communication and expression (Fig. 7B; Supplementary Fig. S8A–S8C).

Consistent with cell-cell communication results, *in vivo* VEGFA activity, defined from Vegfa regulon using NetBID2 algorithm (25) and SJARACNe (50) derived regulon from scRNA-seq of m.CR tumors, and expression were increased in GPR65 KO relative to m.CR tumors (Fig. 7C). Further, mice receiving GPR65 KO tumors had substantially increased serum concentrations of VEGFA compared with mice with m.CR tumors after CAR T-cell treatment (Fig. 7D). *In vitro*, increased VEGFA expression in GPR65 KO tumors was confirmed by Western blotting of cultured cell lysates (Fig. 7E).

To determine whether VEGFA derived from GPR65 KO tumors contributes to CAR T-cell therapy resistance, we generated VEGFA KO cell lines from both m.CR and GPR65 KO cells (Supplementary Fig. S8D). m.CR, VEGFA KO, GPR65 KO, or GPR65 VEGFA double knockout (DKO) tumors were engrafted into immunocompetent mice, followed by treatment with CAR T cells. Mice receiving m.CR and VEGFA KO tumors showed complete responses to CAR T-cell therapy (Fig. 7F and G). As expected, CAR T cells were unable to control GPR65 KO tumor cells. Although the deletion of tumor VEGFA in DKO tumors restored CAR T susceptibility and extended survival, the response was incomplete; after an initial remission, DKO tumors relapsed. Consistently, DKO tumors had fewer tumor cells and macrophage numbers compared with GPR65 KO tumors 4 days after treatment (Fig. 7H and I). Together, these results supported a contribution of increased VEGFA expression from GPR65 KO tumors to CAR T-cell therapy resistance.

### Anti-VEGFA Sensitizes GPR65 KO Tumors to CAR T-cell Therapy

Anti-VEGFA antibodies are clinically available and widely used to treat several solid tumors (51–54). Considering that VEGFA deficiency in DKO tumors bestowed CAR T-cell susceptibility, we further examined whether pharmacologic inhibition of VEGFA would equivalently rescue CAR T sensitivity in GPR65 KO tumors (Supplementary Fig. S9A). Indeed, the administration of CAR T cells and anti-VEGFA antibody significantly prolonged survival and reduced GPR65 KO tumor burden compared with mice receiving isotype control (no anti-VEGFA). Mice bearing m.CR tumors treated with anti-VEGFA remained responsive to CAR T therapy (Supplementary Fig. S9B–S9E). Similar to the DKO tumors, the response of GPR65 KO tumors was incomplete, with mice ultimately succumbing to the tumor. This suggests that anti-VEGFA blockade in combination with CAR T cells is an effective strategy to diminish GPR65-associated resistance to CAR T-cell therapy.

### GPR65 KO Upregulates VEGFA in Tumor Cells via FOXO1 Network

To unbiasedly understand the induction of tumor VEGFA expression via GPR65 signaling, we first constructed a CAR T-cell treatment–specific tumor cell interactome from scRNA-seq data of m.CR tumors using SJARACNe (50) and then performed differential activity (DA) and DE analysis using NetBID2 (Supplementary Fig. S10A; ref. 25). This identified *Foxo1* among the top three TFs upregulated in GPR65 KO compared with m.CR tumor cells (Supplementary Fig. S10B and S10C; Supplementary Table S5). Consistently, increased FOXO1 activity, although not mRNA expression, was identified in GPR65 KO cells (Supplementary Fig. S10D). A heatmap of *Foxo1* target genes inferred by SJARACNe (50) further highlighted the increased FOXO1 activity in GPR65 KO tumors (Supplementary Fig. S10E and S10F). Elevated FOXO1 was confirmed in GPR65 KO cells by immunoblot (Supplementary Fig. S10G). Increased FOXO1 activity in GPR65 KO tumors may be associated with increased levels of VEGFA, as FOXO1 has been shown to induce VEGFA expression in some systems (55). To confirm the role of FOXO1 in VEGFA expression, we generated FOXO1 KO clones of m.CR and GPR65 KO tumors and measured VEGFA levels in culture lysates. VEGFA was, as expected, elevated in GPR65 KO compared with m.CR tumor cells and was significantly diminished after FOXO1 KO (Supplementary Fig. S10H). These findings support a role for FOXO1 in the elevated production of VEGFA by GPR65 KO cells.

## Discussion

Despite the remarkable success of CAR T-cell therapy in the treatment of CD19^+^ hematologic tumors (56), 30% to 50% of patients remain unresponsive or relapse within a year (57). Resistance of these tumors to CAR T-cell therapy due to T-cell exhaustion is well characterized (29). However, the influence of intratumoral heterogeneity and tumor–TME interactions on CAR T-cell response is poorly understood (46, 47). In this study, we demonstrate how B-ALL programming can alter the TME and confer therapy resistance and indicate how this may be therapeutically overcome.

Using an unbiased screen, we identify GPR65 as a determinant associated with CAR T-cell as well as blinatumomab engager therapy response in patients with B-ALL and in *BCR-ABL*–transformed B-ALL cells in an immunocompetent C57BL/6 mouse system. GPR65 is a member of the class A orphan GPCR family that acts as an extracellular pH sensor (16, 17, 58, 59). Although described as an oncogene in solid tumors (18) and a tumor suppressor in hematologic malignancies (19, 60), GPR65 has not previously been associated with susceptibility to immunotherapy.

GPR65 deficiency did not influence several known modalities leading to CAR T-cell resistance. CD19 antigen expression was unchanged, and there was no evidence of altered T-cell activation, exhaustion, or cytokine secretion *in vitro*. To the contrary, *in vivo* CAR T-cell numbers were increased and demonstrated elevated activation and functional markers, consistent with the increased tumor burden present in mice bearing GPR65 KO tumors. In addition, the role of an acidic pH in local invasion and metastasis has been proposed for solid tumors (61). Our observation that GPR65 KO tumor–bearing mice display a higher disease burden in all organs, suggesting potential extramedullary disease, is consistent with the published findings. However, further research is required to verify these alternatives and fully understand the implications of acidic TMEs on B-ALL progression. *In vitro* analyses of GPR65 KO and m.CR tumor cells demonstrated equivalent sensitivity to killing, arguing against an intrinsic resistance to lysis among GPR65 KO tumor cells. Likewise, CAR T cells isolated from within GPR65 KO or m.CR tumors and retransferred into mice with susceptible GPR65 KO CAR T tumors demonstrated equivalent tumoricidal potential. These data point to an indirect pathway for GPR65 KO tumor resistance and, by exclusion, implicate the TME as responsible.

The TME is a heterogeneous mixture of cells and may include an abundant population of tumor-associated macrophages (refs. 45, 62, 63), in which the polarization state can foster T-cell exclusion and immune privilege (41, 64). M2-like phenotypes are anti-inflammatory and associated with tumor progression, whereas M1-like phenotypes are inflammatory and associated with antitumor responses (65–67). We identify both an increase in the overall macrophage number and a shift to an M2 phenotype in B-ALL with loss of GPR65. Through cross-species analysis of tumor-associated macrophages, we identified similar M2 polarization in CD19^+^ relapse patients after CD19 CAR T-cell therapy. This further supports our findings and the human disease relevance of GPR65 KO–driven CAR T-cell therapy resistance. Although we cannot discern the distinct impact of heterogeneous macrophage subsets, their numbers, and polarization on CAR T-cell therapy resistance, the complete reversal of GPR65 KO tumor resistance upon macrophage depletion confirms their critical role in CAR T-cell therapy resistance. These findings align with our observations that GPR65 deficiency acts through the TME and does not cause tumor intrinsic resistance to CAR T-cell therapy.

Multiple cues prompt the recruitment and differentiation of macrophages in the TME (42). Unbiased cell–cell communication analyses of tumor cells with host macrophages and monocytes pinpointed VEGFA as a major communication hub specifically upregulated in GPR65 KO tumors. There is now a strong and growing literature on VEGF’s impacts on myeloid cells and as a therapeutic target. This literature is consistent with our findings in identifying VEGFA as a modulator of myeloid function and immune response (68–70). Likewise, our scRNA-seq analyses of enriched F4/80^+^ macrophages isolated from the TME demonstrate heterogeneity in the macrophage population. This functional diversity of macrophages in the TME is gaining recognition for its role in promotion of tumor growth, lineage plasticity, invasion, remodeling of the extracellular matrix, and cross-talk with endothelial cells, mesenchymal stromal cells, and other immune cells (71). A causal link between this macrophage heterogeneity and the loss of GPR65, along with its consequent effects on the TME-dependent resistance, needs further investigation. Tumor-derived VEGFA is known to recruit macrophages to tissue, contribute to M2 polarization, and can foster tumor growth (72–74). We hypothesize that the increased number of macrophages, their heterogeneity, and their M2 polarization leads to CAR T therapy resistance. That VEGFA and GPR65 DKO tumors show restoration of CAR T susceptibility and that this reduces the TME macrophage number and tumor burden implicate tumor-produced VEGFA as a key mediator of CAR T resistance in the B-ALL system. Rational combination of anti-VEGFA and CAR T-cell therapy shows similar effects, further establishing the feasibility of reprogramming the TME to support CAR T function using existing immunotherapies. Both antibody- and signaling-based VEGF inhibitors are clinically approved and the standard of care for some solid tumors (51–54). That anti-VEGF therapy may be similarly effective in a hematologic malignancy residing in highly vascularized organs suggests that VEGF functions in a manner unrelated to its angiogenic effects. Indeed, VEGFA recruits macrophages and also promotes M2 specification (73). Nevertheless, additional mechanistic dissection of VEGF and macrophage polarization here is important and may lead to additional insights on targets restricting CAR T responses in hematologic malignancies.

Although VEGFA is identified as one mechanism of tumor resistance to therapy, other pathways seem to be involved. Indeed, we identified multiple cell communication pathways distinguishing CAR T cell–sensitive m.CR and resistant GPR65 KO tumors that may contribute to macrophage abundance and polarization. It is also noteworthy that although macrophages and VEGF play an important role in GPR65 KO–mediated primary resistance to CAR T therapy, the distinct *in vivo* kinetics of GPR65-high m.CR and GPR65-low m.PR tumor cells studied here and the ultimate CD19^−^ relapse observed in CAR T–treated m.PR tumor–engrafted mice illustrate the complexity of individual tumors and the TME and their mechanisms of resistance. Taken together, these observations point to the potential for alternative pathways to additionally contribute to the resistance observed in GPR65-low CAR T, blinatumomab, and other immunotherapy scenarios for B-ALL. Consequently, more in-depth investigations utilizing single-cell sequencing are needed to fully elucidate the changes within the TME at low GPR65 levels.

GPR65 may have multiple effects on cellular physiology, highlighted by our hidden driver analysis. Included among these, FOXO1 was identified as one of the top TFs driving differences in the transcriptional profile of GPR65 KO and m.CR tumors. FOXO1 has been linked to VEGFA expression in other cell types (55). We confirm that FOXO1 influences VEGFA production in both GPR65 KO and m.CR B-ALL although a pathway linking GPR65, FOXO1, and VEGFA remains to be fully resolved.

Our findings demonstrate the influence of subtle differences in the transcriptional wiring of identical tumor subtypes in causing marked changes in the TME and responsiveness to CAR T immunotherapy. This can help explain how a subset of patients fail immunotherapy. Much as immune escape through tumor CD19 loss and lineage switch may conceal tumor cells from CAR T, our findings demonstrate that tumor-induced shifts in the microenvironment can markedly alter the ability of CAR T cells to effectively engage and destroy tumor cells. This can occur in the absence of CAR T-cell exhaustion or intrinsic dysfunction and without evidence for tumor-intrinsic resistance to lysis. Furthermore, the identification of a GPCR as a primary mediator of this phenotype suggests additional therapeutic opportunities directly targeting this key signaling molecule. GPCRs are readily targeted through small molecules, and the development of GPR65 activators may conceivably provide therapeutic opportunities in patients receiving CAR T cells in which tumors possess low levels of GPR65.

## Methods

### Mice

C57BL/6 mice were obtained from The Jackson Laboratory (strain 000664). Human CD19 CAR transgenic mice were generated as previously described (7). All studies used 8- to 12-week-old female mice housed in an American Association for Accreditation of Laboratory Animal Care–accredited facility. Experimental protocols were approved by the St. Jude Children’s Research Hospital Animal Care and Use Committee in accordance with NIH guidelines.

### Cell Lines

C56BL/6J luciferase-expressing *Arf*^−/−^ BCR-ABL1^+^Ph^+^ progenitor B-ALL cells were transduced with MSCV-hCD19-IRES-RFP and cloned as previously described to isolate m.PR and m.CR clones (7, 20). In brief, bone marrow cells from the *Arf*^−/−^ CD45.1 host were transduced with BCR-ABL1-luc and injected retro-orbitally into a CD45.2 host and monitored for disease progression. Animals that developed the disease were sacrificed, and CD45.2^+^CD19^+^ cells were sorted, confirmed for BCR-ABL1-luc expression, transduced with MSCV-hCD19-IRES-RFP, and RFP sorted for single clones. The cells were cultured at 37°C in 8% CO_2_ and RPMI medium with 100 U/mL penicillin/streptomycin, 100 µg/mL sodium pyruvate, 1× minimum essential medium (MEM) Non-essential amino acids (NEAA), 1× GlutaMAX, 10 mmol/L HEPES, and 1 mmol/L β-mercaptoethanol (all from Gibco) and 10% FBS (Biowest). GPR65 KO, VEGFA-KO, and FOXO1-KO cell lines were generated using CRISPR/Cas9. Gene knockout was performed by electroporation with a ribonucleoprotein (RNP) complex. The RNP complex was prepared by mixing 450 pmol (in 9 µL volume) of a precomplexed guide(g)RNA/tracrRNA duplex and 180 pmol (in 6 µL volume) of the Cas9 protein (UC Berkeley) for 10 minutes at room temperature. For clones generated with more than one gRNA, equimolar concentrations of each were combined to total 450 pmol. The cells were resuspended at 0.5 × 10^6^ cells per 20 µL of P4 primary cell nucleofection solution (Lonza), and 5 µL of the RNP complex was added. The cell/RNP mixture was incubated for 2 minutes at room temperature, electroporated (4D-Nucleofector Core Unit, Program DI-100, Lonza), and transferred to prewarmed RPMI media. gRNA sequences were GPR65 g1: 5′-AGACTATTACTGCTAGAAGT-3′, GPR65 g2: 5′-TCATCCATGCATTTAGAGAG-3′, GPR65 g3: 5′-ACATTGTATGACTCCTATGT-3′, GPR65 g4: 5′-ACTGTTCGTCTTTAAATCAG-3′ GPR65 g5: 5′-GGAACAAATAGTGTTCGAGG-3′, VEGFA g1: 5′-GAAGATGTACTCTATCTCGT-3′, VEGFA g2: 5′- ATTCACATCTGCTGTGCTGT-3′, Foxo1 g1: 5′-CCAGGCTCGCCGCGGCGTCGNGG-3′, and Foxo1 g2: 5′-GGCCGCCAACCCCGACGCCGNGG-3′. All cell lines used in this study were authenticated using short tandem repeat profiling. Short tandem repeat analysis was performed by amplifying polymorphic microsatellite markers, including D1Mit-chromosome-1, D3Mit-chromosome-3, D6Mit-chromosome-6, D8Mit-chromosome-8, D10Mit-chromosome-10, and D13Mit-chromosome-13, and comparing the profiles with in-house authentication records to confirm cell line identity and strain background. Additionally, species-specific PCR assays targeting conserved murine gene regions (e.g., cytochrome c oxidase I and 18S rRNA) were used to verify the absence of cross-species contamination. *Mycoplasma* testing was performed using the MycoStrip Mycoplasma Detection Kit (InvivoGen) Alert assay with routine screenings conducted (e.g., monthly and quarterly). The most recent test was performed on September 24, 2024, and no contamination was detected. If a cell line tested positive, it was either discarded or treated following standard decontamination protocols before reevaluation. Cells were used for experiments between 3 and 4 passages after thawing or collection, typically within 2 to 4 weeks of culture. The biological sex of the cell lines used in this study is unknown.

### CAR T Cells

T cells were enriched from the spleen and lymph nodes of CD19 CAR transgenic mice by magnetic-activated cell sorting using the Pan T cell Isolation Kit (Miltenyi) and resuspended in complete RPMI medium with 10% FBS. Then, the T cells were activated with 5 µg/mL plate-bound anti-mouse CD3 (clone) and CD28 (clone) and 20 U/mL recombinant human IL-2 (PeproTech) for 2 days at 37°C in 5% CO_2_. The cells were transferred to new plates and cultured in 20 U/mL recombinant human IL-2, 5 ng/mL recombinant human IL-7 (PeproTech), and 25 ng/mL recombinant human IL-15 (PeproTech) for 4 to 5 days prior to experimental use.

### *In Vivo* Survival, Imaging, and Analysis

Mice were injected intravenously with 1 × 10^6^ luciferase-expressing hCD19^+^ B-ALL tumor cells followed 7 days later with 10 × 10^6^ of activated CAR T cells or PBS (retro-orbital). For survival experiments, tumor growth was monitored by bioluminescence imaging. Mice received 200 µL of 15 µg/mL D-luciferin (PerkinElmer) i.p., and images were acquired after 5 minutes using a Xenogen IVIS-200 (PerkinElmer). For other experiments, mice were sacrificed at predetermined time points, and organs were harvested and processed into single-cell suspensions for analysis. Macrophage depletion experiments used the same protocol, except beginning 5 days after tumor injection mice received a depleting dose of 400 μg of either αCSF1R (Bio X Cell, cat. #BE0213, RRID: AB_2687699) or rat IgG2a isotype control (Bio X Cell, cat. #BE0089, RRID: AB_1107769) by intraperitoneal injection, followed by maintenance doses of 100 μg three times per week for 28 days. For CAR T-cell retransfer experiments, mice received 1 × 10^6^ m.CR tumor cells intravenously, followed 7 days later by 2 × 10^6^ CAR T cells, either freshly expanded or sorted from the spleens of mice with m.CR or GPR65 KO tumors 3 days after treatment. The VEGFA neutralization experiment used the same protocol. The treatment consisted of four rounds on days 3, 6, 8, and 10 after tumor injection, with mice receiving 5 mg/kg of either VEGFA neutralizing antibodies (BioLegend, cat. #512810, RRID: AB_2814440) or control antibodies (Bio X Cell, cat. #BE0089, RRID: AB_1107769) by intraperitoneal injection.

### Flow Cytometry

For surface staining, single-cell suspensions were incubated with Ghost Dye BV510 or Ghost Dye Red 710 in PBS to stain dead cells. Samples were washed and incubated in PBS with 2% FBS, and the following fluorochrome-conjugated anti-mouse antibodies were used: CD3 (BioLegend, cat. #100336, RRID: AB_11203705), CD8 (BioLegend, cat. #100779, RRID: AB_2832268), CD19 (BioLegend, cat. #302279, RRID: AB_2894448), B220 (BioLegend, cat. #103243, RRID: AB_11203907), NK1.1 (BD Biosciences, cat. #557391, RRID: AB_396674), Ly6G (BioLegend, cat. #127608, RRID: AB_1186099), Ly6C (BioLegend, cat. #128008, RRID: AB_1186132), Ter119 (BioLegend, cat. #116208, RRID: AB_313708), CD11b (BioLegend, cat. #101212, RRID: AB_312794), F4/80 (BioLegend, cat. #123132, RRID: AB_10901171), Thy1.1 (BioLegend, cat. #202506, RRID: AB_492882), CD45 (BioLegend, cat. #103168, RRID: AB_2832301), PD-1 (BioLegend, cat. #135203, RRID: AB_1877086), Lag3 (Thermo Fisher Scientific, cat. #16-2231-81, RRID: AB_494125), CD69 (BioLegend, cat. #104513, RRID: AB_492844), CD25 (BioLegend, cat. #102072, RRID: AB_2894645), CD44 (BioLegend, cat. #103026, RRID: AB_493713), and CD62L (BioLegend, cat. #104459, RRID: AB_2832327). Anti-human CD19 (BioLegend, cat. #302279, RRID: AB_2894448) was used to stain the tumor cell hCD19 antigen. For intracellular staining, cells were cultured for 4 to 6 hours in complete RPMI with Cell Stimulation Cocktail (eBioscience), surface stained, fixed, permeabilized, and stained with the following fluorochrome-conjugated anti-mouse antibodies: IFNγ (BioLegend, cat. #505827, RRID: AB_2295769), TNFα (BioLegend, cat. #506351, RRID: AB_2888813), IL-2 (BioLegend, cat. #503823, RRID: AB_2123675), and GZMB (BioLegend, cat. #515408, RRID: AB_2562196). For intranuclear staining, the cells were surface stained, followed by fixation and permeabilization with the FOXP3/transcription factor staining buffer set (eBioscience) and stained for BCL2. Samples were analyzed on a BD LSRFortessa (BD Biosciences) and sorted using a FACS Aria (BD Biosciences). Analyses were performed using FlowJo software (BD Biosciences).

### Bulk RNA-seq

RNA was isolated from *in vitro* cell cultures or sorted cells using the RNeasy plus mini kit (Qiagen). Three biological replicates were submitted for RNA-seq. For total RNA-seq, library construction from RNA used the Illumina TrueSeq stranded mRNA library prep kit, and sequencing was performed using the HiSeq or NovaSeq platforms (2 × 101-bp paired-end reads). Analyses were performed using previously established procedures (75). Gene expression was quantified as fragments per kilobase of transcript per million mapped reads using RSEM v1.2.28PP with the GRCm38.p6 reference genome and annotation file (Gencode v30).

### Cross-Species Integration, Gene Ranking, and CAR T Response Signature Generation Using RNA-seq Data of Patient B-ALL and Mouse B-ALL Samples Treated with CAR T

Human and mouse B-ALL tumor RNA-seq data were log2count per million (CPM)-normalized, followed by DE analysis using the NetBID2 function getDE.limma.2G(), comparing h.CR versus h.NR for human and m.CR versus m.PR for mouse. Two results were merged to only keep genes overlapped in both species. To define integration anchors, genes were first filtered using abs (*Z*-score) >1.96, and then only those genes with directional consistency across both human and mouse (sign of log_2_ fold change the same in both human and mouse) were kept. Final feature numbers were made equal for both human and mouse using minimum [length (human, mouse)] ranked by abs (log_2_ fold change). The two-tailed Fisher exact test was performed to test the statistical significance of overlap between mouse and human anchor features. The human log_2_CPM–normalized and mouse log_2_CPM–normalized matrix of anchor features were *Z*-scaled and merged, and principal component analysis was performed using the R package PCAtools (v3.18) to jointly visualize human and mouse B-ALL tumors. The PC1 score was used as the CAR T therapy response score to perform ROC analysis for evaluation of the CAR T therapy response score to predict the response of patients with B-ALL and mice to CAR T-cell therapy using the R package pROC (v1.18.5). Final gene ranking between human and mouse responders versus nonresponders was derived by combining *Z*-scores using the Stouffer method. Alternatively, NetBID2 analysis was performed as described (75). Briefly, 27 B-ALL samples from GSE130663 (h.NR = 8 and h.CR = 19) and 39 adult B-ALL samples (responders = 19 and nonresponders = 20) from the European Genome-phenome Archive (EGA; accession EGAS00001004027) were analyzed using NetBID2 (v2.0.3). The GPR65 regulon (50 genes) was constructed by SJARACNe (v0.2.1) using B-ALL–specific RNA-seq profiles (*N* = 1,988) from patients with B-ALL (76). The “cal.Activity” function (method of “weightedmean”) in NetBID2 was employed to infer the activity of GPR65 for each patient. Statistical significance was evaluated using the Wilcoxon rank-sum test comparing responder and nonresponder groups.

### scRNA-seq

Harvested splenocytes were stained and sorted to isolate m.CR or GPR65 KO tumor cells (RFP^+^) and host immune cells (RFP^−^Thy1.1^−^CD45.2^+^) from mice with m.CR or GPR65 KO tumors prior to or 4 days after CAR T treatment. Tumor and host cells from CAR T–untreated mice were mixed at a 1:1 ratio and analyzed together. Tumor and host cells from treated mice were run independently. Each sample contained a total of 200,000 cells. The cells were counted using AO/PI stain (Luna Cell fl Counter, Logos Biosystems), and 6,000 cells were targeted from each sample using the 10X Chromium Next GEM Single Cell 5′ Reagent kits v2 (Dual Index). The cells were partitioned into droplets and barcoded using the Chromium Controller with Chip K. Reverse transcription, cDNA amplification, T-cell receptor amplification, fragmentation, and adapter and sample index ligation were all performed following the 10X CG000331 Rev C protocol. cDNA and libraries were quantified using the High Sense DNA chip on the Agilent Bioanalyzer. Libraries were pooled and sequenced on a NovaSeq 6000 using paired-end reads with the following configuration: read 1: 26 nucleotides, index 1: 10 nucleotides, index 2: 10 nucleotides, and read 2: 90 nucleotides. Cell Ranger (v6.0.0) Single-Cell software suite (10X Genomics) was used to process raw Illumina NovaSeq 6000 sequencing data. Demultiplexing, alignment (GRCm38.p6), and barcode processing generated gene–cell matrices used for downstream analysis. The cells with low or high unique molecular identifier counts were filtered, as well as those with high mitochondrial gene reads (>7.5%). The TME was annotated using known markers from literature, and plasma cells were removed from all downstream analyses. Tumor cells were annotated using inferCNV (​^31^–^34^​).

### scRNA-seq after Enrichment of F4/80^+^ Macrophages

Splenocytes were harvested from GPR65 KO or m.CR tumor–bearing mice on day 7 or 11, before and after CAR T-cell treatment, and enriched for macrophages using F4/80 staining by FACS. Macrophages, non-macrophage immune cells, and RFP-expressing tumor cells were isolated and mixed at a ratio of 3:2:1 (RFP neg CD45.2^+^F4/80^+^ macrophages/RFP neg Thy1.1 neg F4/80 neg CD45.2^+^ TME/RFP^+^ tumor cells). The cells were counted using AO/PI stain on the Luna fl Cell Counter (Logos Biosystems). Tumor and host cells from individual treated and untreated mice were fixed separately using the 10X Genomics CG000478 Demonstrated Protocol Cell & Nuclei Fixation Chromium Fixed RNA Profiling Rev B. After fixation, individual samples were counted and barcoded with unique sample barcodes during probe hybridization. Uniquely barcoded samples were counted using trypan blue stain, then pooled in equal numbers following the 10X Genomics CG000565 Chromium Fixed RNA Profiling Multiplexed Samples Pooling Workbook Rev B, and washed, and 8,000 cells were targeted from each sample to be partitioned into GEMS on Chip Q using the 10X Genomics Chromium Fixed RNA Profiling kit. Partitioned cells underwent barcoding, reverse transcription, cDNA amplification, and sample index ligation following the 10X Genomics CG000527 Chromium Fixed RNA Profiling Multiplexed Samples User Guide Rev D. Libraries were quantified using the D5000 kit on the Agilent TapeStation and Illumina MiSeq platform. Quantified libraries were then sequenced using an S2 flow cell on the Illumina NovaSeq 6000.

### Clustering Analysis, DE, GSEA, and Data Visualization

Clustering analysis was performed with scMINER (v0.1.0). In brief, MICA was initiated with mode “ge” and parameter “ar = 4.0” using scMINER (v0.1.0). Subclustering of mouse macrophages was performed using the first 10 principal components and a Louvain resolution of 0.2 after subsetting host cells positive for macrophage markers *Adgre1* and *Cd68.* DE analysis between GPR65 KO tumor–derived versus m.CR tumor–derived mouse macrophages and CD19^+^ relapse–derived versus h.CR patient bone marrow–derived macrophages was performed using the FindMarkers() function in Seurat (v4.3.0). GSEA of mouse and human macrophages was performed using C7 gene sets from MsigDB (v7.5.1) using NetBID2 functions cal.Activity.GS() and getDE.limma.2G(), respectively. Human bone marrow scRNA-seq data were downloaded from EGA accession number EGAD00001010018, and macrophages were annotated from three responders and two CD19^+^ relapse patients using *CD14* and *CD68* expression, followed by scMINER and GSEA as described above.

### Cell–Cell Communication Analysis

For cell–cell communication analysis between m.CR or GPR65 KO tumors and host macrophages, LIANA (v0.1.12) was used to perform CellChat analysis on the Ensemble mouse ligand–receptor database using the function liana_wrap() with method = “call_cellchat” and resource = “MouseConcensus” for m.CR and GPR65 KO tumors separately. The computeCommunProb() function was used with the default parameter to quantify ligand–receptor interaction probability. Communication gain in GPR65 KO tumors was defined using the setdiff() function on ligand–receptor pairing between GPR65 KO and m.CR groups after filtering statistically significant cell–cell communication using *P* < 0.05. Finally, cell–cell communication between m.CR and GPR65 KO tumors was visualized using the chordDiagramFromDataFrame() function from the R package circlize (v0.4.15).

### Hidden Driver Analysis from scRNA-Seq Data

A CAR T-cell treatment–specific m.CR tumor TF interactome was reconstructed using SJARACNe (v0.2.1) using the parameters “−*n* = 100” and “−pc = 0.01” from gene expression profiles of 2,726 m.CR tumor cells. The resulting interactome contained 13,773 nodes and 294,367 edges. To identify hidden drivers of GPR65 KO tumors, the activity of 1,035 TFs from the SJARACNe-generated interactome was calculated using the NetBID2 function cal.activity(), es.method = “weightedmean” for GPR65 KO tumor cells (*N* = 1,389), and m.CR tumor cells (*N* = 2,726), followed by DA and DE analysis using the NetBID2 function getDE.limma.2G() using the activity matrix (calculated above) or log_2_CPM-normalized scRNA-seq expression matrix. The drivers were ranked by average activity, and the top hidden drivers comparing GPR65 KO versus m.CR tumors were filtered using *Z*-score DA >1.96 and *Z*-score DE <0.

### qRT-PCR

RNA was isolated from *in vitro* cell cultures in triplicate using the RNeasy plus mini kit (Qiagen) and subjected to cDNA synthesis with the High-Capacity cDNA Reverse Transcription Kit (Thermo Fisher Scientific) according to the manufacturer’s instructions. qRT-PCR was performed using SYBR Green master mix (Thermo Fisher Scientific) on a QuantStudio 7 Pro system (Thermo Fisher Scientific). Relative gene expression was calculated using the 2^−ΔΔCT^ method with 18S RNA as the reference gene. Primer sequences were as follows: *Gpr65* F: 5′-ATGGCGATGAACAGCATGTG-3′, *Gpr65* R: 5′-ACGCATAAAGATCCGATGTTGG-3′, *Vegfa* F: 5′-GCACATAGAGAGAATGAGCTTCC-3′, *Vegfa* R: 5′-CTCCGCTCTGAACAAGGCT-3′.

### Immunoblotting

Cells were lysed in NP-40 lysis buffer (Research Products International); whole-cell lysate protein concentrations were determined by Bicinchoninic Acid (BCA) assay (Thermo Fisher Scientific), and 20 µg of protein was subjected to SDS-PAGE. Gels were transferred to polyvinylidene difluoride (PVDF) membranes and immunoblotted with primary antibodies against mouse pCREB (Abcam), total CREB (Cell Signaling Technology), VEGFA (Proteintech), actin (MilliporeSigma), and FOXO1 (Cell Signaling Technology). Luminata Crescendo Western HRP substrate (Millipore) was added to immunoblots, and chemiluminescence was detected with a ChemiDoc Touch Imaging System (Bio-Rad). For phospho-protein immunoblots, phosphorylated protein was detected first, and blots were stripped with Restore PLUS Western blot stripping buffer (Thermo Fisher Scientific) prior to total protein detection.

### cAMP Assay

m.PR, m.CR, or GPR65 KO tumor cells (2 × 10^6^) from *in vitro* culture were analyzed with the cAMP Direct Immunoassay Kit (Abcam) per the manufacturer’s protocol in triplicate.

### *In Vitro* Tumor Cell Expansion Assay

m.CR or GPR65 KO tumor cells were seeded in triplicate in a 96-well plate at 5 × 10^3^ cells/well. The cells were counted manually using a hemocytometer, and the total number of live cells per well was calculated over 6 days of culture.

### Data Availability

Bulk and scRNA-seq data generated by this study have been deposited in the Gene Expression Omnibus (GEO) database under accession number GSE266166. RNA-seq data of 1,988 patients with B-ALL were downloaded from the EGA under accession number EGAS00001003266. Bulk bone marrow RNA-seq profiles of B-ALL tumors from 27 patients (19 responders and eight nonresponders) were downloaded from GSE130663. Bulk RNA-seq data of 39 adult B-ALL samples (19 responders and 20 nonresponders) were downloaded from the EGA under accession number EGAS00001004027. scRNA-seq data of unsorted bone marrow from five patients (three responders and two CD19^+^ relapses) were downloaded from the EGA under accession number EGAD00001010018. All the codes used to perform the analyses are available upon request. Any additional information required to reanalyze the data reported in this article is available from the lead contact (J. Yu) upon request.

## Supplementary Material

Supplementary Tables S1-S5 Table S1: Differential expression and Activity analysis of human (h.CR vs h.NR) and mouse (m.CR vs m.PR) B-ALL tumor samples. The colum is ranked by combined Z-score of human and mouse DE results. Table S2: Functional enrichment analysis upregulated and downregulated genes in m.CR vs m.PR tumors RNA-seq. Table S3: Differential Expression analysis results of CART T cells treated with No tumor, CR tumors or GPR65 KO tumors. Table S4: Differential Expression analysis of GPR65 KO vs m.CR tumor cells from Day 7 Untreated, Day 11 Untreated and Day 11 CAR-T cell treated tumors. Table S5: NetBID2 Hidden driver analysis of 1035 TF comparing CAR T Treated GPR65 KO and CR tumors.

Supplementary Figure S1 Figure S1 shows that GPR65 is a biomarker of B-ALL immunotherapy response

Supplementary Figure S2 Figure S2 shows the strategy to generate GPR65 clones.

Supplementary Figure S3 Figure S3 shows that GPR65 KO mediated CAR-T resistance is independent of antigen expression and CAR-T cell expansion.

Supplementary Figure S4 Figure S4 shows CAR-T cell function remains unimpaired in GPR65 KO TME.

Supplementary Figure S5 Figure S5 is the scRNA-seq experiment design to evaluate tumors and TME.

Supplementary Figure S6 Figure S6 shows characterization of mouse and human B-ALL TME.

Supplementary Figure S7 Figure S7 shows macrophage depletion and CAR-T cell therapy regimen.

Supplementary Figure S8 Figure S8 shows cell-cell communication analysis of tumors, macrophages, and monocytes.

Supplementary Figure S9 Figure S9 shows that anti-VEGFA sensitizes GPR65 KO tumors to CAR-T cell therapy.

Supplementary Figure S10 Figure S10 shows that GPR65 KO upregulates VEGFA in tumor cells via FOXO1 network.
