# Supplementary material for: GPR65 Inactivation in Tumor Cells Drives Antigen-Independent CAR T-cell Resistance via Macrophage Remodeling
Source: Cancer Discov. 2025 Feb 25;15(5):1018–36. doi: 10.1158/2159-8290.CD-24-0841 (PMC12046320; doi:10.1158/2159-8290.CD-24-0841)
Supplement: Supplementary Figure S1 — Figure S1 shows that GPR65 is a biomarker of B-ALL immunotherapy response [file cd-24-0841_supplementary_figure_s1_suppsf1.docx]

**
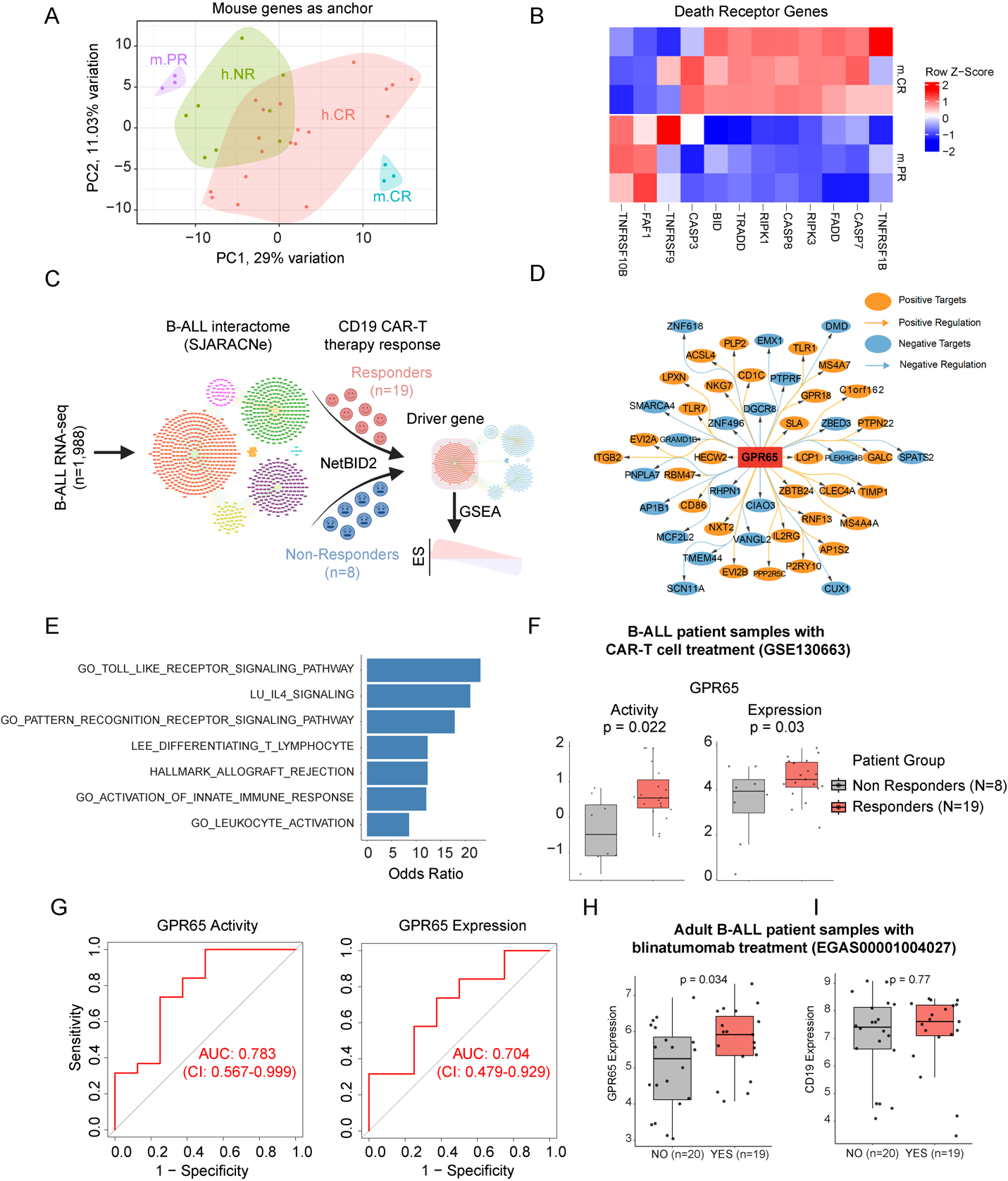
**

**Supplementary Figure S1:** **GPR65 is a biomarker of B-ALL immunotherapy response** (A) PCA plot integrating human and mouse RNA-seq profiles performed using expression of 307 mouse CAR-T response signature genes. (B) Heatmap of RNA-seq of m.CR and m.PR tumor cells for 12 differentially expressed death receptor genes identified from B-ALL RNA-seq of 27 patients by Singh et. al. (C) Schematic diagram of NetBID2 analysis workflow to infer GPR65 regulon from 1,988 B-ALL patients RNA-seq profiles. (D) GPR65 target genes reverse-engineered from B-ALL-specific RNA-seq profiles of 1,988 B-ALL patients using the SJARACNe algorithm. Orange indicates predicted positively regulated and blue negatively regulated GPR65 target genes. (E) Functional enrichment analysis of GPR65 target genes (50 genes) performed using merged Hallmark, Canonical pathways, CGP chemical and genetic perturbations, and Gene Ontology pathways from MsigDB (v.7.5.1). Statistical significance was calculated using Fisher’s Exact Test, and statistically significant pathways filtered using BH corrected p < 0.05 were plotted. (F) Boxplot of GPR65 activity (left) and expression (right) calculated using the NetBID2 algorithm from RNA-seq profiles of B-ALL patients treated with CAR-T cells. Responders (N=19) are patients those who achieved complete remission (> 1 year), and Non-Responders (N=8) are patients who did not respond to CAR-T cell therapy and had no evidence of CD19 antigen loss. P-value was estimated using Wilcoxon rank-sum test. Boxplot shows summary of the data interims of the minimum, maximum, sample median, and the first and third quartiles. (G) Receiver operator characteristic (ROC) curve plot for evaluation of GPR65 activity (left) and expression (right) to predict the response of B-ALL patients to CD19 CAR-T cell therapy. (H) Boxplot of GPR65 expression in adult B-ALL patients treated with blinatumomab. Responders (N=19) are patients those who achieved complete remission by morphology using standard International Working Group criteria, and Non-Responders (N=20) are patients who did not respond to blinatumomab therapy and had no evidence of CD19 antigen loss. (I) Corresponding boxplot of CD19 expression in adult B-ALL patients treated with blinatumomab. (H-I) P-value was estimated using Wilcoxon Rank Sum Test. Boxplots show summary of the data interims of the minimum, maximum, sample median, and the first and third quartiles.
