# Supplementary material for: GPR65 Inactivation in Tumor Cells Drives Antigen-Independent CAR T-cell Resistance via Macrophage Remodeling
Source: Cancer Discov. 2025 Feb 25;15(5):1018–36. doi: 10.1158/2159-8290.CD-24-0841 (PMC12046320; doi:10.1158/2159-8290.CD-24-0841)
Supplement: Supplementary Figure S2 — Figure S2 shows the strategy to generate GPR65 clones. [file cd-24-0841_supplementary_figure_s2_suppsf2.docx]

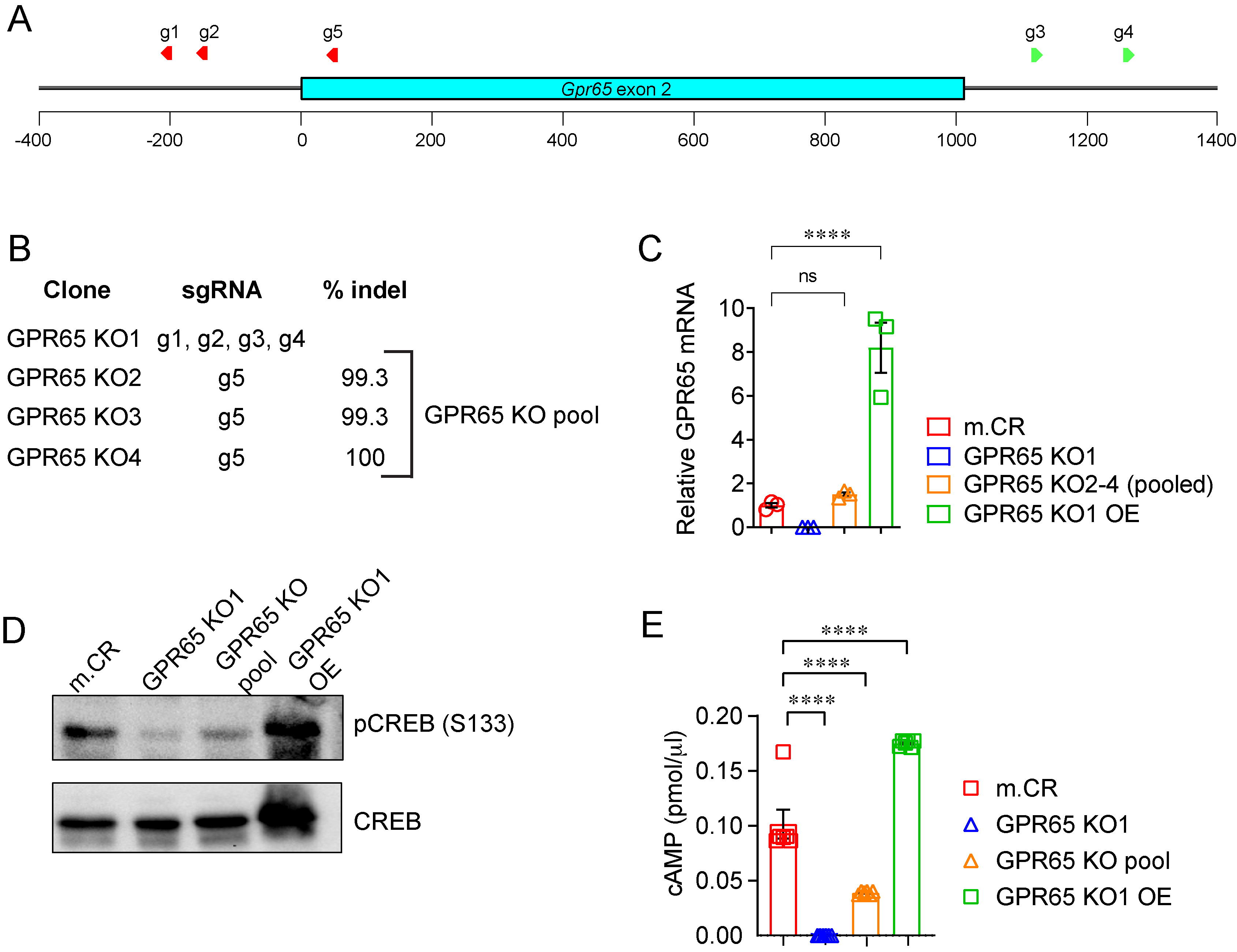


**Supplementary Figure S2: Strategy to generate GPR65 KO clones** (A) Schematic drawing of *Gpr65* exon 2 showing binding sites of gRNA used to generate GPR65 KO clones. (B) List of GPR65 KO cell lines showing gRNA used to generate each clone and % indel from TIDE analysis. (C) qRT-PCR analysis of relative *Gpr65* levels in m.CR, GPR65 KO1, GPR65 KO pool, and GPR65 KO1 OE cell lines. Representative of two experiments, n=3 replicates per group. Significance was determined by unpaired t-test. (D) Immunoblot showing pCREB and total CREB protein levels in m.CR, GPR65 KO1, GPR65 KO pool, and GPR65 KO1 OE cell lines. (E) cAMP concentration in m.CR, GPR65 KO1, GPR65 KO pool, and GPR65 KO1 OE cell lines. n=6 replicates per group. Representative of 2 experiments. Significance was determined by one-way ANOVA with Tukey’s post-test. All error bars represent mean + SEM. ****p < 0.0001.
