# Supplementary material for: GPR65 Inactivation in Tumor Cells Drives Antigen-Independent CAR T-cell Resistance via Macrophage Remodeling
Source: Cancer Discov. 2025 Feb 25;15(5):1018–36. doi: 10.1158/2159-8290.CD-24-0841 (PMC12046320; doi:10.1158/2159-8290.CD-24-0841)
Supplement: Supplementary Figure S3 — Figure S3 shows that GPR65 KO mediated CAR-T resistance is independent of antigen expression and CAR-T cell expansion. [file cd-24-0841_supplementary_figure_s3_suppsf3.docx]

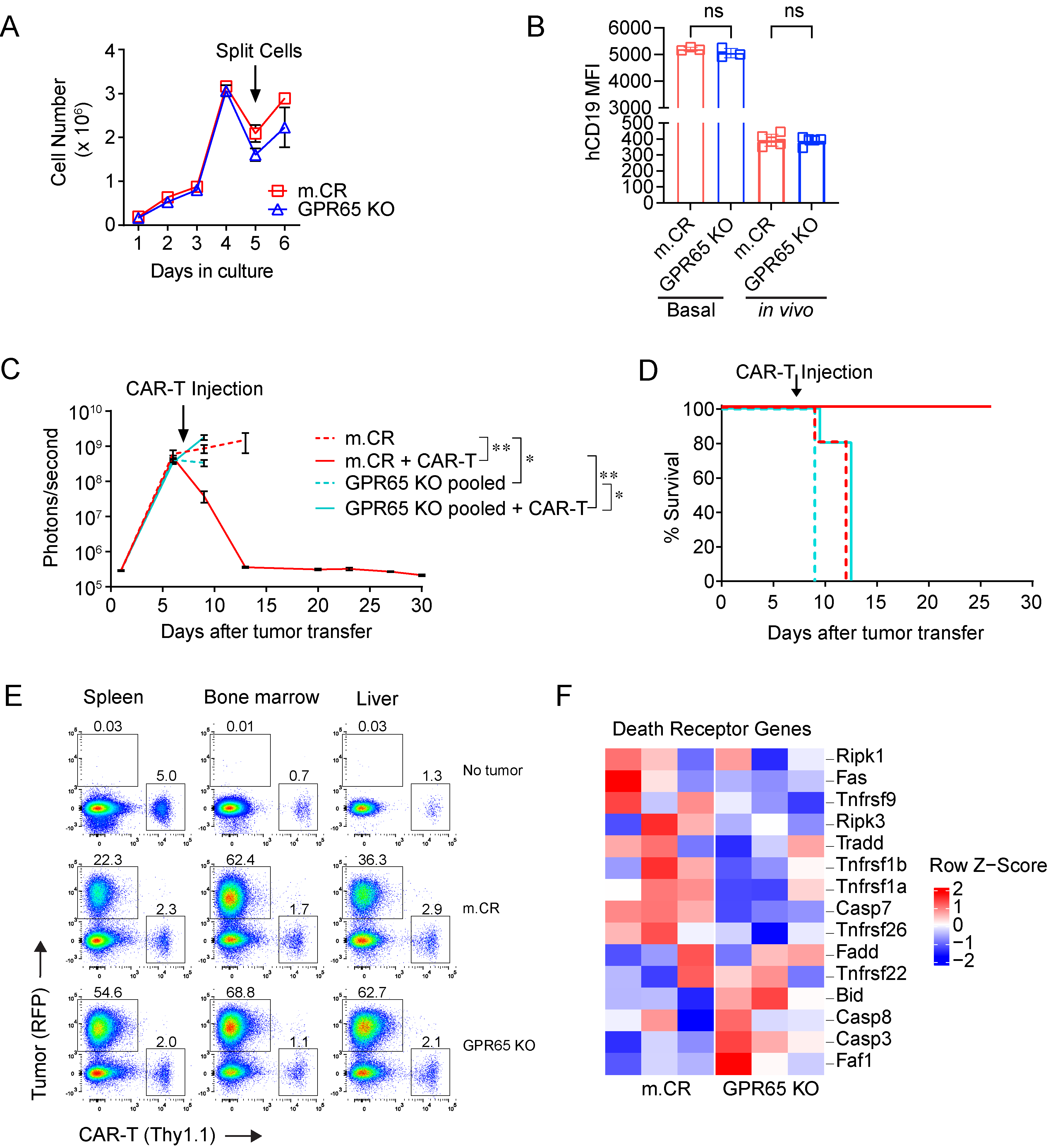


**Supplementary Figure S3: GPR65 KO mediated CAR-T resistance is independent of antigen expression and CAR-T cell expansion** (A) Expansion of equivalently plated m.CR or GPR65 KO tumor cells over 6 days of *in vitro* culture. (B) Surface expression of human CD19 on m.CR and GPR65 KO tumor cells from *in vitro* culture (basal) and *in vivo*. *In vivo* hCD19 expression was measured at day 7 preceding CAR-T infusion. N=5 mice per group. (C-D) Mice received 1x10^6^ m.CR or GPR65 KO pool tumor cells, followed by treatment with 10x10^6^ CAR-T cells or PBS 7 days later. (C) Bioluminescence imaging showing tumor growth. Representative of two experiments, n=5 mice per group. (D) Kaplan-Meier survival curves. Significance was determined by log-rank (Mantel-Cox) test. Representative of two experiments, n=5 mice per group. (E) Spleen, bone marrow, and liver were harvested 4 days after CAR-T treatment and analyzed by flow cytometry. Representative dot plots showing tumor and CAR-T cell gating. (F) Heatmap of RNA-seq of m.CR and GPR65 KO tumor cells for 15 differentially expressed death receptor genes identified from B-ALL RNA-seq of 27 patients by Singh et. al. Significance was determined by one-way ANOVA with Tukey’s post-test for multiple comparisons. All error bars represent mean + SEM. *p < 0.05; **p < 0.01; ***p < 0.001.
