# Supplementary material for: GPR65 Inactivation in Tumor Cells Drives Antigen-Independent CAR T-cell Resistance via Macrophage Remodeling
Source: Cancer Discov. 2025 Feb 25;15(5):1018–36. doi: 10.1158/2159-8290.CD-24-0841 (PMC12046320; doi:10.1158/2159-8290.CD-24-0841)
Supplement: Supplementary Figure S4 — Figure S4 shows CAR-T cell function remains unimpaired in GPR65 KO TME. [file cd-24-0841_supplementary_figure_s4_suppsf4.docx]

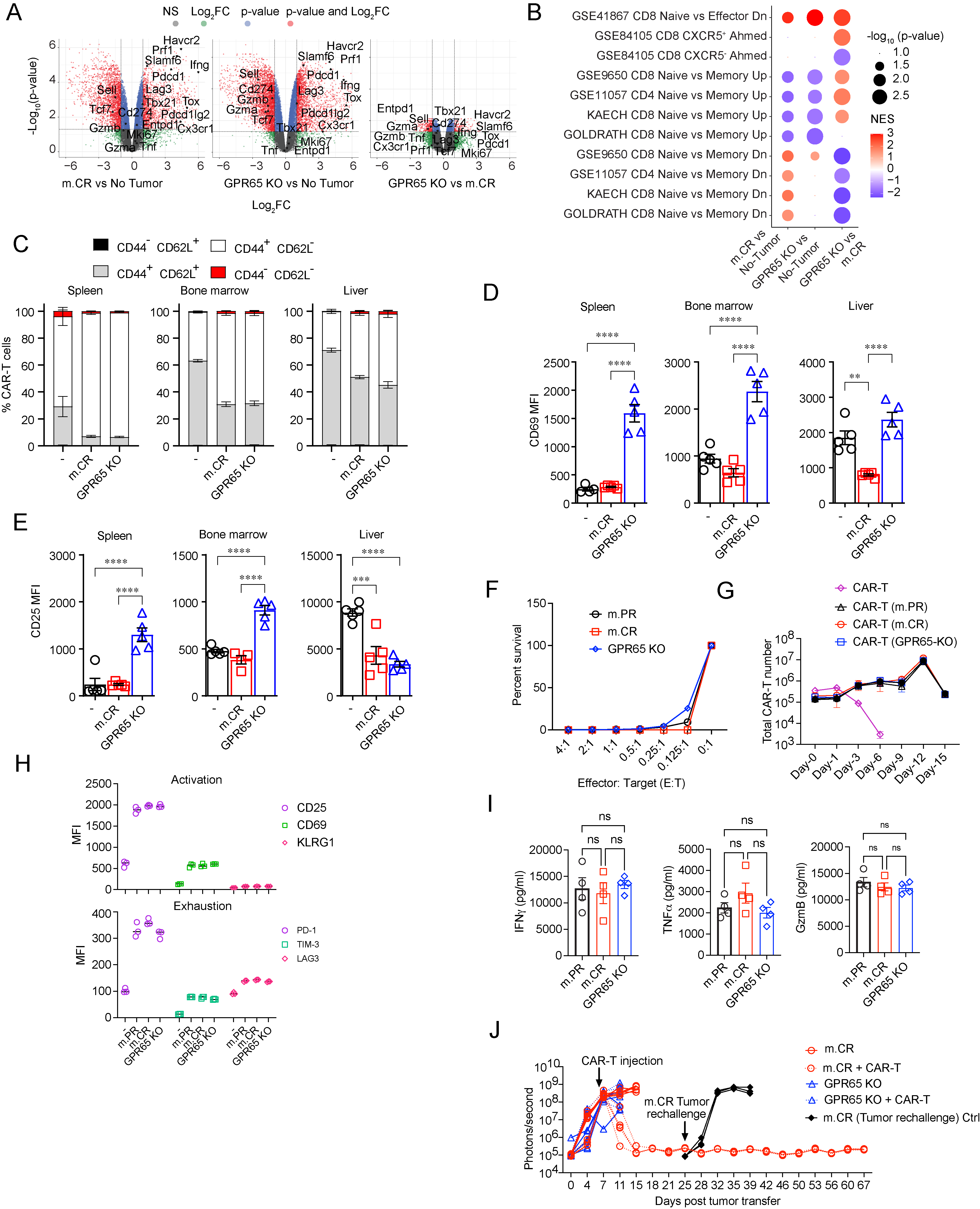


**Supplementary Figure S4: CAR-T cell function remain unimpaired in GPR65 KO TME** (A) Volcano plot of differential expression analysis comparing CAR-T cells in m.CR vs No-tumor, GPR65 KO vs No-Tumor and GPR65 KO vs m.CR. Genes associated with T cell effector or exhaustion are highlighted. Red color indicates genes that pass statistical significance threshold of |Log_2_FC| > 1 and BH corrected p-value < 0.05, blue color indicates genes that pass statistical significance threshold of BH corrected p-value < 0.05, green color indicates genes that pass |Log_2_FC| threshold > 1 and grey color indicates genes that did not show significant change based on above mentioned criteria. (B) Bubble plot of GSEA analysis performed using differentially expressed genes from (A) on curated gene sets of T cell effector and memory signatures. Red and blue color indicates positive or negative enrichment score, and size of the bubble indicates statistical significance score (-log_10_(p-value)). (C-E) Mice received 1x10^6^ m.CR or GPR65 KO tumor cells, or PBS followed by treatment with 10x10^6^ CAR-T cells 7 days later. Organs were harvested 4 days after CAR-T treatment for flow cytometric analysis. (C) Percent of CD44 and CD62L populations among CAR-T cells in spleen, bone marrow, and liver. (D) MFI of CD69 and (E) CD25 in CAR-T cells from spleen, bone marrow, and liver. Representative of two experiments. Significance was determined by one-way ANOVA with Tukey’s post-test for multiple comparisons. (F) Percentage of live m.PR, m.CR or GPR65 KO tumor cells after 48 hours of co-culture with CAR-T cells at the indicated effector to target ratios. Representative of three experiments, n=2 replicates. (G) *In vitro* repetitive co-culture assay showing CAR-T cell fold expansion performed using 5x10^5^ m.PR, m.CR or GPR65 KO tumor cells and 5x10^5^ CAR-T cells at 1:1 ratio. Representative of two experiments. (H) MFI of activation markers (CD25, CD69), terminal differentiation marker (KLRG1) and inhibitory markers (PD-1, TIM-3, and LAG3) in CAR-T cells co-cultured with m.PR, m.CR or GPR65 KO tumors in repetitive stimulation assay at third stimulation (day3). (I) *In vitro* cytokine levels of IFNγ, TNFα, and GZMB measured using ELISA on supernatant collected from repetitive stimulation assay at day 3. Significance was determined by one-way ANOVA with Tukey’s post-test for multiple comparisons. (J) *In vivo* tumor bioluminescence imaging of rechallenge experiment. Mice received 1x10^6^ m.CR or GPR65 KO tumor cells (day 0), and 10x10^6^ CAR-T cells or PBS 7 days later. m.CR engrafted mice that responded completely received a secondary challenge of 1x10^6^ m.CR tumor cells and tumor burden was monitored. A second group of naïve mice were injected with 1x10^6^ m.CR tumors as a control group (black lines). n = 5 mice per group. All error bars represent mean + SEM. **p < 0.01; ***p < 0.001; ****p < 0.0001.
