# Supplementary material for: GPR65 Inactivation in Tumor Cells Drives Antigen-Independent CAR T-cell Resistance via Macrophage Remodeling
Source: Cancer Discov. 2025 Feb 25;15(5):1018–36. doi: 10.1158/2159-8290.CD-24-0841 (PMC12046320; doi:10.1158/2159-8290.CD-24-0841)
Supplement: Supplementary Figure S5 — Figure S5 is the scRNA-seq experiment design to evaluate tumors and TME. [file cd-24-0841_supplementary_figure_s5_suppsf5.docx]

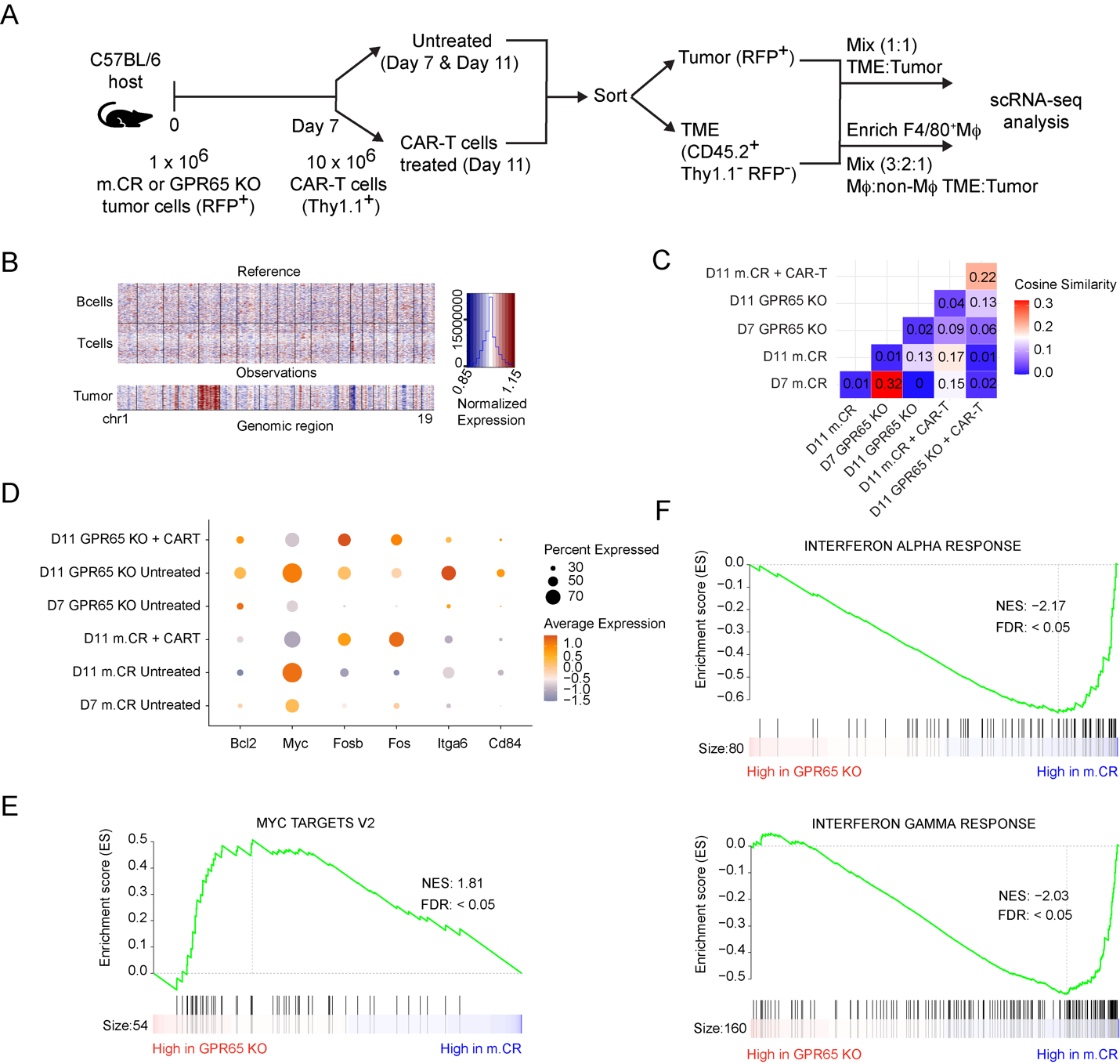


**Supplementary Figure S5: scRNA-seq experiment design to evaluate tumors and TME** (A) Schematic showing the experimental design for single-cell RNA sequencing. (B) InferCNV heatmap of copy number alternation for annotation of tumor cells with gain in copy number compared to B cells and T cells as references. (C) Heatmap of cosine similarity analysis performed on scRNA-seq of m.CR and GPR65 KO tumors before and after CAR-T cell therapy. Gradient of blue indicates low similarity and gradient of red indicates high similarity using midpoint, (0.16) as reference. (D) Bubble plot highlighting key differentially expressed genes in CAR-T cell treated and untreated GPR65 KO vs. m.CR tumors at day 7 and day 11. (E) Representative GSEA plot showing enrichment of Myc gene sets from MsigDB (v.7.5.1) for CAR-T cell treated m.CR or GPR65 KO tumors at day 11. (F) Representative GSEA plot showing enrichment of interferon gene sets from MsigDB (v.7.5.1) for CAR-T cell treated m.CR or GPR65 KO tumors at day 11.
