# Supplementary material for: GPR65 Inactivation in Tumor Cells Drives Antigen-Independent CAR T-cell Resistance via Macrophage Remodeling
Source: Cancer Discov. 2025 Feb 25;15(5):1018–36. doi: 10.1158/2159-8290.CD-24-0841 (PMC12046320; doi:10.1158/2159-8290.CD-24-0841)
Supplement: Supplementary Figure S6 — Figure S6 shows characterization of mouse and human B-ALL TME. [file cd-24-0841_supplementary_figure_s6_suppsf6.docx]

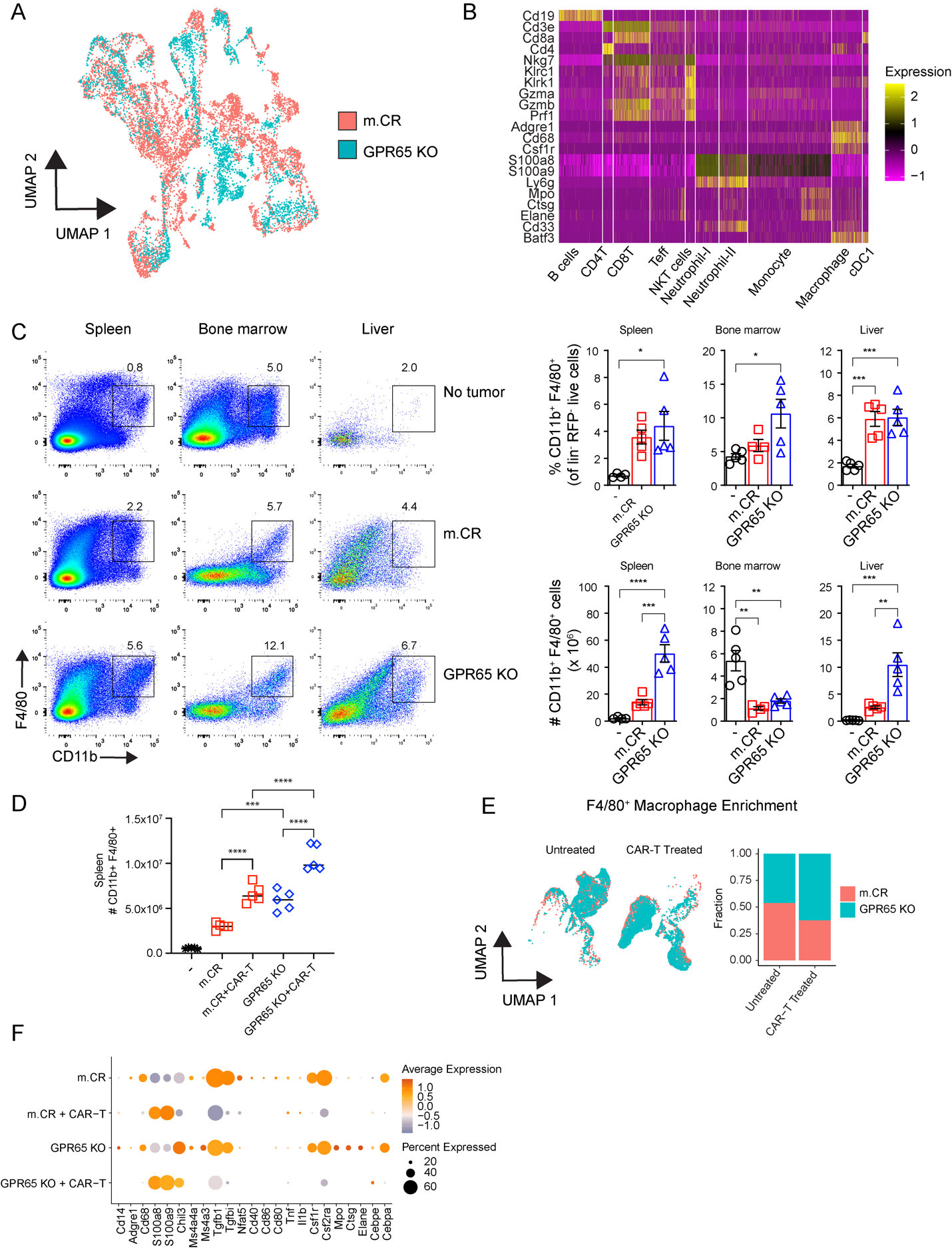


**Supplementary Figure S6:** **Characterization of mouse and human B-ALL TME** (A) Single-cell RNA-seq UMAP plot of host TME from mice bearing m.CR or GPR65 KO tumors. Representative of single-cell RNA-seq of tumor and TME performed at tumor:TME ratio of 1:1 (CAR-T cell treated GPR65 KO or m.CR tumor bearing mice). (B) Heatmap of marker genes of cells used to annotate TME populations from mice bearing m.CR or GPR65 KO tumors (C) Representative dot plots (left) and summary data (right) showing the percentage and total number of Lin^–^RFP^–^CD11b^+^F4/80^+^ cells from indicated organs four days after CAR-T treatment. Representative of three experiments, n=3-5 mice per group. Significance was determined by one-way ANOVA with Tukey’s post-test for multiple comparisons. (D) Dot plot of CD11b^+^ F4/80^+^ macrophages numbers from spleen of mice engrafted with m.CR or GPR65 KO tumors before and after CAR-T cell therapy. Representative of four experiments, n=5 mice per group. Significance was determined by one-way ANOVA with Tukey’s post-test for multiple comparisons. All error bars represent mean + SEM. * p < 0.05; ** p < 0.01; *** p < 0.001; **** p < 0.0001. (E) UMAP plot of F4/80^+^ cells from untreated and CAR-T treated mice engrafted with either m.CR or GPR65 KO tumors (left), and bar plot of fraction of F4/80^+^ cells before and after CAR-T treatment in mice engrafted with m.CR or GPR65 KO tumors (right). Representative of single-cell RNA-seq of F4/80^+^ enriched macrophages from TME of m.CR or GPR65 KO tumors at Untreated or CAR-T treated condition. (F) Bubble plot of expression of macrophage and myeloid genes used to annotate F4/80^+^ cells before and after CAR-T treatment in mice engrafted with m.CR or GPR65 KO tumors. Representative of single-cell RNA-seq of F4/80^+^ enriched macrophages from TME of m.CR or GPR65 KO tumors at Untreated or CAR-T treated condition.
