# Supplementary material for: GPR65 Inactivation in Tumor Cells Drives Antigen-Independent CAR T-cell Resistance via Macrophage Remodeling
Source: Cancer Discov. 2025 Feb 25;15(5):1018–36. doi: 10.1158/2159-8290.CD-24-0841 (PMC12046320; doi:10.1158/2159-8290.CD-24-0841)
Supplement: Supplementary Figure S7 — Figure S7 shows macrophage depletion and CAR-T cell therapy regimen. [file cd-24-0841_supplementary_figure_s7_suppsf7.docx]

**
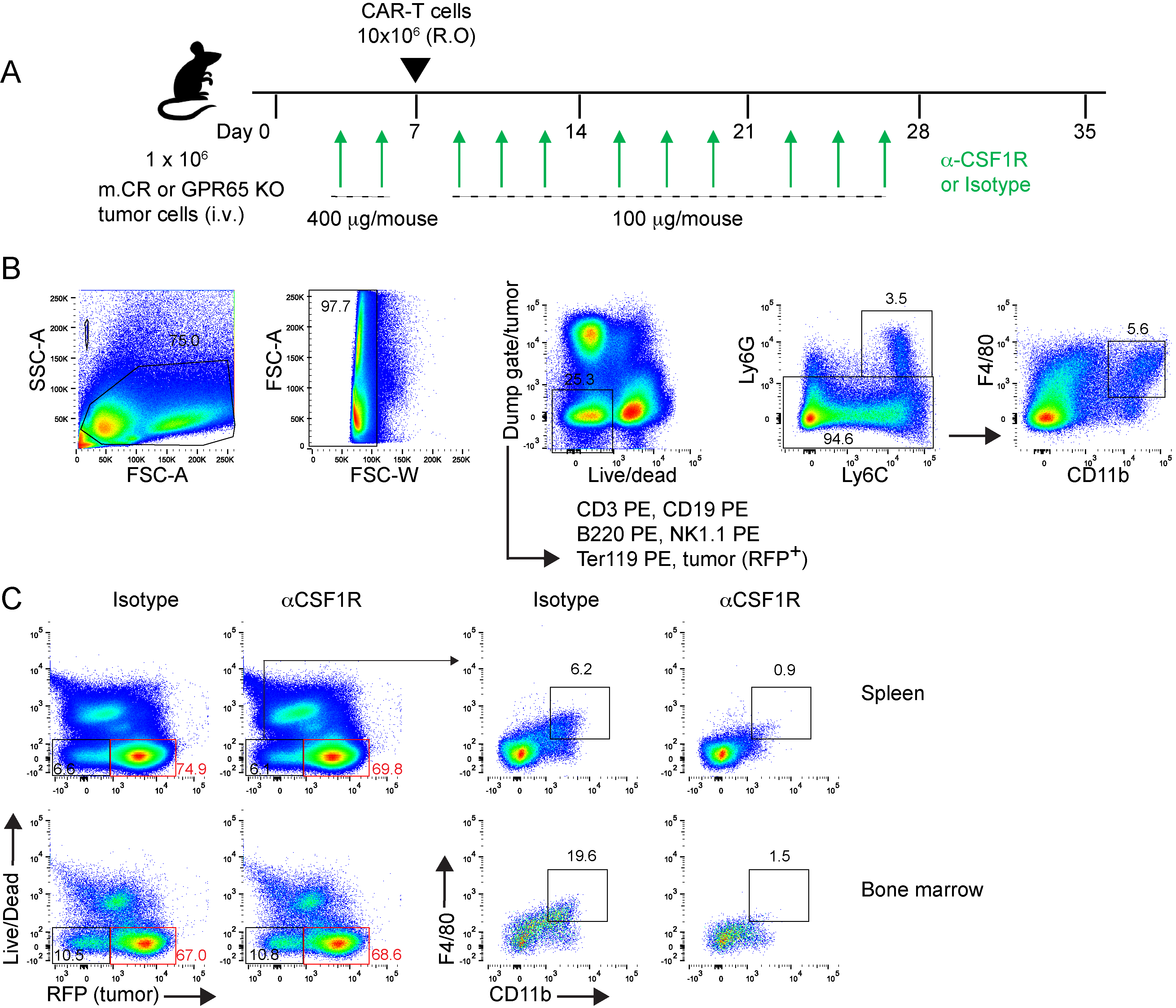
**

**Supplementary Figure S7:** **Macrophage depletion and CAR-T cell therapy regimen** (A) Schematic of macrophage depletion experiment. Mice received m.CR or GPR65 KO tumors followed by a depleting dose of 400μg per mouse of either αCSF1R or isotype control five days later. Maintenance doses of αCSF1R or isotype control at 100μg per mouse were given three times per week for up to 28 days after tumor transfer. All groups received CAR-T cell treatment seven days after tumor transfer. (B) Macrophage gating strategy is shown. Cells expressing RFP (tumor) or selected lineage markers (CD3, CD19, B220, Ter119, NK1.1) were excluded using a dump gate, followed by exclusion of Ly6C^+^Ly6G^+^ neutrophils. (C) Depletion of splenic and bone marrow macrophages with αCSF1R is shown. Mice received GPR65 KO tumors, followed by αCSF1R or isotype antibody 5 days later. Spleen and bone marrow samples were analyzed after a further 2 days. Dot plots showing gating to exclude dead and tumor cells (left) followed by CD11b and F4/80 gating for macrophages (right).
