# Supplementary material for: GPR65 Inactivation in Tumor Cells Drives Antigen-Independent CAR T-cell Resistance via Macrophage Remodeling
Source: Cancer Discov. 2025 Feb 25;15(5):1018–36. doi: 10.1158/2159-8290.CD-24-0841 (PMC12046320; doi:10.1158/2159-8290.CD-24-0841)
Supplement: Supplementary Figure S8 — Figure S8 shows cell-cell communication analysis of tumors, macrophages, and monocytes. [file cd-24-0841_supplementary_figure_s8_suppsf8.docx]

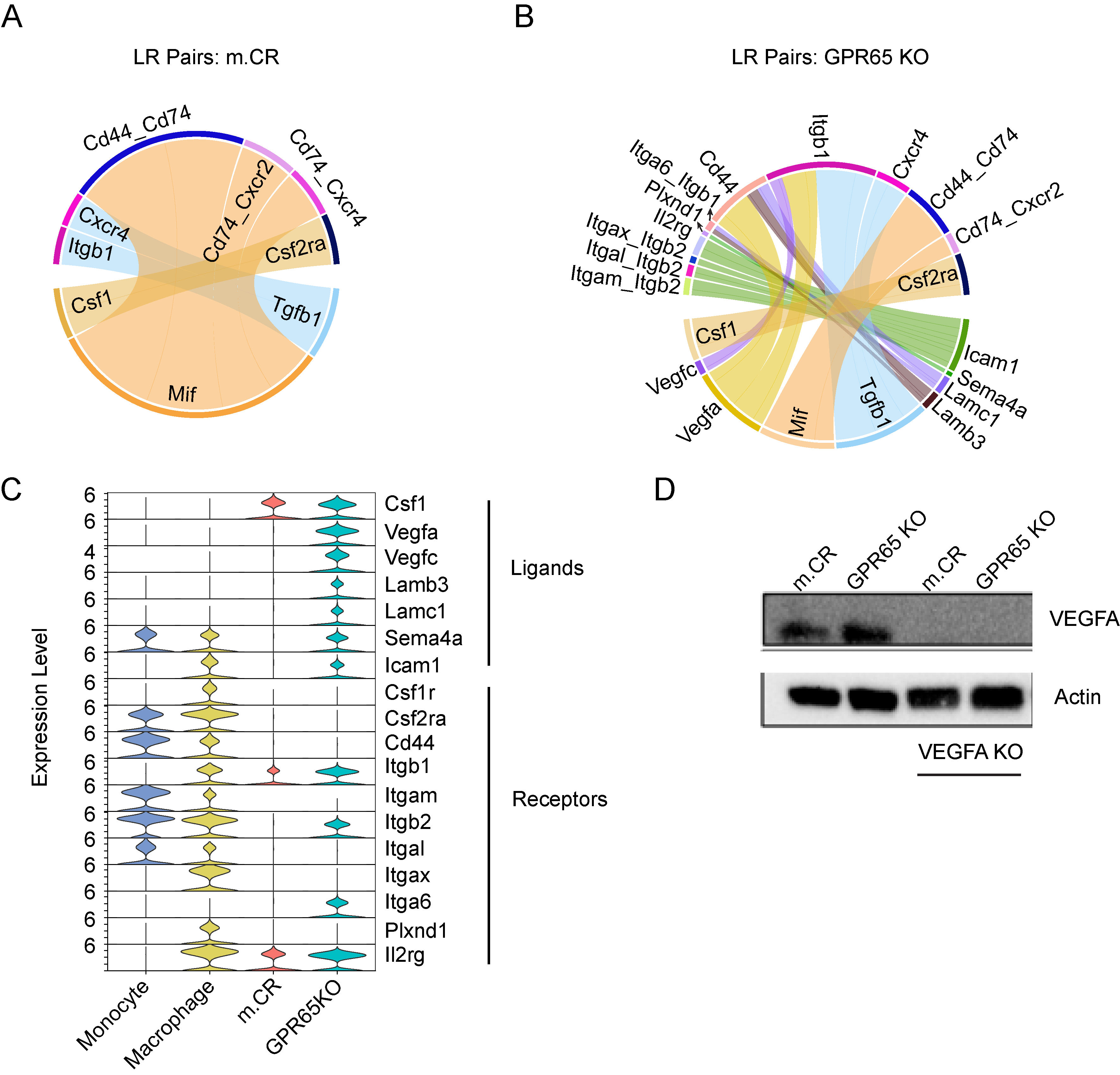


**Supplementary Figure S8:** **Cell-Cell communication analysis of tumors, macrophages and monocytes** (A) Chord diagram highlighting all significant ligand receptor pairs between m.CR tumors, host macrophages, and host monocytes of mice treated with CAR-T cells. Significant ligand receptor pairs were filtered at p-value < 0.05 from the output of CellChat computeCommunProb() function. (B) Chord diagram highlighting all ligand receptor pairs between GPR65 KO tumors, host macrophage and host monocytes of mice treated with CAR-T cells. Significant ligand receptor pairs were filtered at p-value < 0.05 from the output of CellChat computeCommunProb() function. (C) Gene expression violin plots of all significant ligand and receptor pairs gained by GPR65 KO tumors from (B) in the single-cell RNA-seq profile of CAR-T treated GPR65KO or m.CR tumors, macrophage, and monocytes. (D) Immunoblot showing VEGFA and Actin proteins levels in m.CR, GPR65 KO, VEGFA KO, and VEGFA and GPR65 DKO tumor cell lysates from culture.
