# Supplementary material for: GPR65 Inactivation in Tumor Cells Drives Antigen-Independent CAR T-cell Resistance via Macrophage Remodeling
Source: Cancer Discov. 2025 Feb 25;15(5):1018–36. doi: 10.1158/2159-8290.CD-24-0841 (PMC12046320; doi:10.1158/2159-8290.CD-24-0841)
Supplement: Supplementary Figure S9 — Figure S9 shows that anti-VEGFA sensitizes GPR65 KO tumors to CAR-T cell therapy. [file cd-24-0841_supplementary_figure_s9_suppsf9.docx]

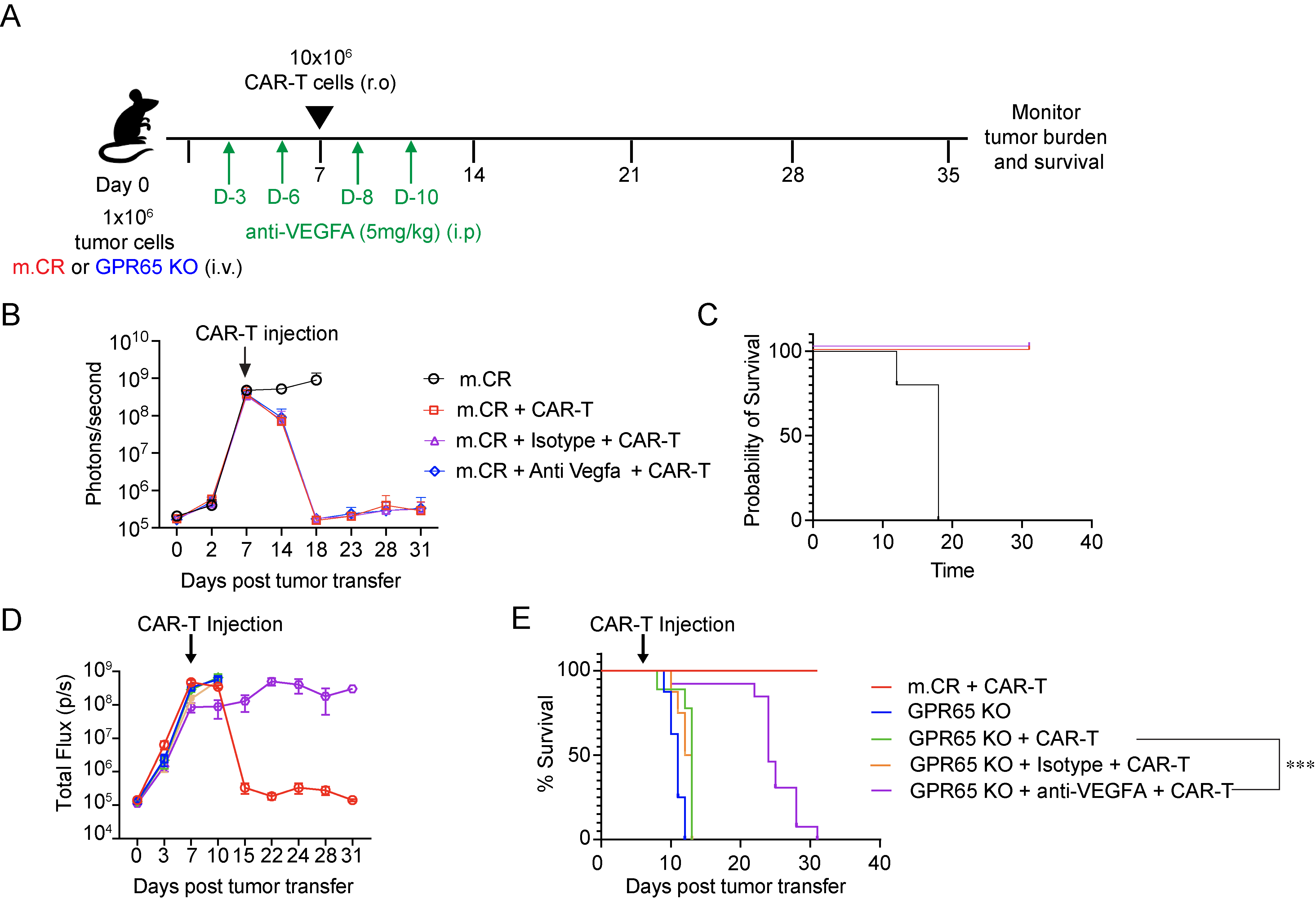


**Supplementary Figure S9: Anti-VEGFA sensitizes GPR65 KO tumors to CAR-T cell therapy** (A) Schematic shows combination regimen for treatment with anti VEGFA and CAR-T cells. (B) Bioluminescence imaging showing tumor growth of m.CR cell with or without anti-Vegfa and CAR-T treatment. Representative of two experiments, n=5 mice per group. (C) Kaplan-Meier survival curves. (D) Bioluminescence imaging showing tumor growth of GPR65 KO cell with or without anti-Vegfa and CAR-T treatment. Representative of two experiments, n=5 mice per group. (E) Kaplan-Meier survival curves. Statistical significance was calculated using log-rank (Mantel-Cox) test. All error bars represent mean + SEM. p < 0.01; ***
