# Supplementary material for: GPR65 Inactivation in Tumor Cells Drives Antigen-Independent CAR T-cell Resistance via Macrophage Remodeling
Source: Cancer Discov. 2025 Feb 25;15(5):1018–36. doi: 10.1158/2159-8290.CD-24-0841 (PMC12046320; doi:10.1158/2159-8290.CD-24-0841)
Supplement: Supplementary Figure S10 — Figure S10 shows that GPR65 KO upregulates VEGFA in tumor cells via FOXO1 network. [file cd-24-0841_supplementary_figure_s10_suppsf10.docx]

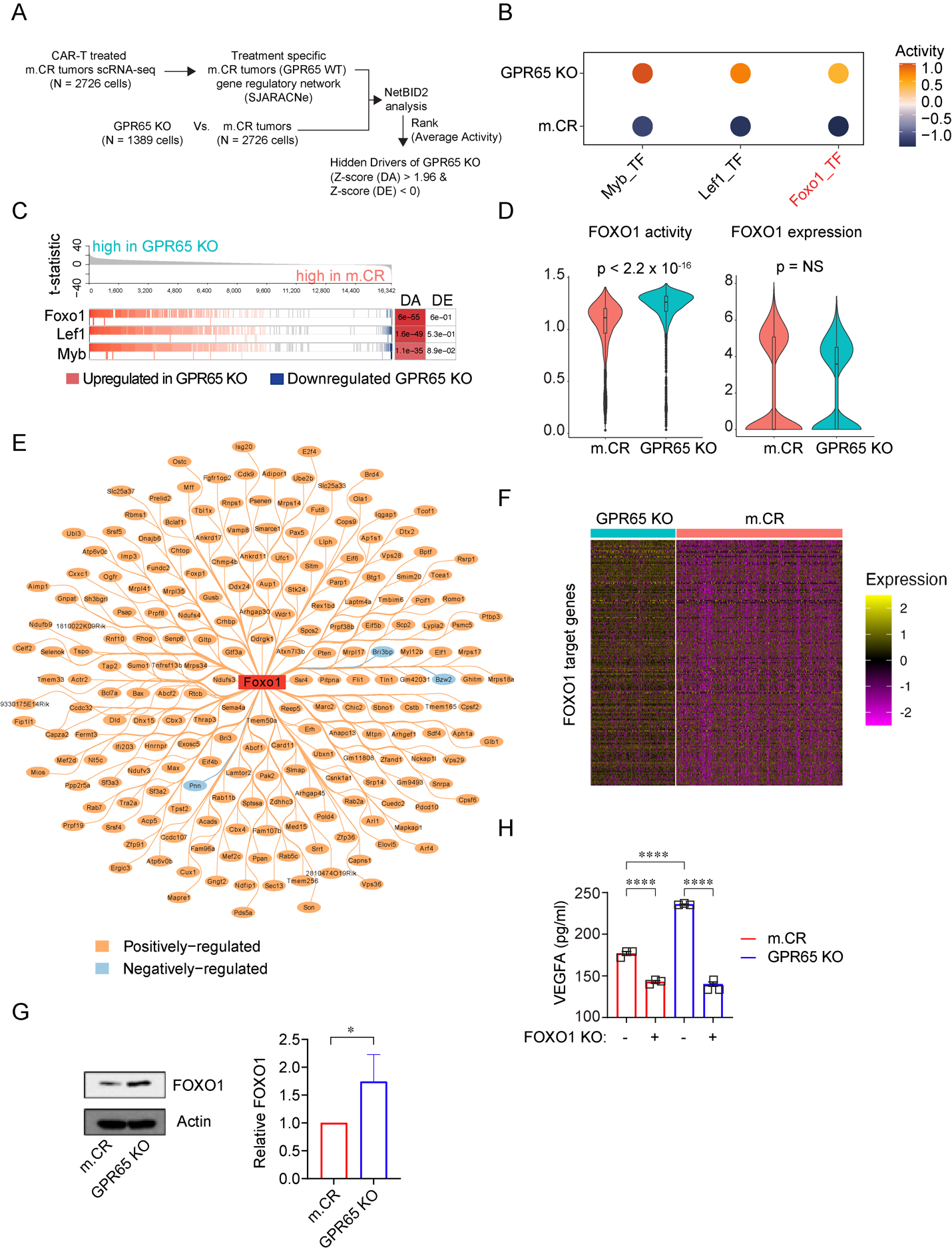


**Supplementary Figure S10:** **GPR65 KO upregulates VEGFA in tumor cells via FOXO1 network** (A) NetBID2 analysis strategy to unbiasedly identify hidden drivers of GPR65 KO tumors driving CAR-T cell therapy resistance via VEGFA. (B) Bubble plot of NetBID2 derived activity of top 3 hidden drivers of GPR65 KO vs. m.CR tumor derived from single-cell RNA-seq data in tumors 4 days after CAR-T cell treatment. Representative of single-cell RNA-seq of tumor and TME performed at tumor:TME ratio of 1:1 (CAR-T cell treated GPR65 KO or CR tumor bearing mice). (C) GSEA of top three hidden driver target genes inferred using SJARACNe comparing GPR65 KO and m.CR tumors after CAR-T cell treatment. (D) NetBID2 derived Foxo1 activity (left) and expression (right) in GPR65 KO and m.CR tumors after CAR-T cell treatment. Statistical significance was calculated using a two-tailed t-test using t.test() function in R. (E) Network plot of Foxo1 regulon inferred using SJARACNe from scRNA-seq expression of m.CR tumors. Orange color represents positively regulated targets, and blue color represents negatively regulated targets. (F) Heatmap of Foxo1 target genes from (E) in GPR65 KO or m.CR tumor cells. (G) Immunoblot of FOXO1 and Actin (left) and its quantification from two experiments (right). (H) VEGFA concentrations in culture medium of m.CR, FOXO1 KO, GPR65 KO, or GPR65 and FOXO1 double KO tumors. Representative of two independent experiments. Significance was determined by one-way ANOVA with Tukey’s post-test for multiple comparisons. All error bars represent mean + SEM. *p < 0.05; **p < 0.01; ***p < 0.001; ****p < 0.0001.
